# Repeat-induced point mutation in *Neurospora crassa* causes the highest known mutation rate and mutational burden of any cellular life

**DOI:** 10.1186/s13059-020-02060-w

**Published:** 2020-06-16

**Authors:** Long Wang, Yingying Sun, Xiaoguang Sun, Luyao Yu, Lan Xue, Zhen He, Ju Huang, Dacheng Tian, Laurence D. Hurst, Sihai Yang

**Affiliations:** 1 State Key Laboratory of Pharmaceutical Biotechnology, School of Life Sciences, Nanjing University, Nanjing, 210023 China; 2 State Key Laboratory for Crop Genetics and Germplasm Enhancement, Nanjing Agricultural University, Nanjing, 210095 China; 3 Department of Biology and Biochemistry, The Milner Centre for Evolution, University of Bath, Bath, UK

**Keywords:** *Neurospora crassa*, Repeat-induced point (RIP) mutation, Mutational burden, Resequencing

## Abstract

**Background:**

Repeat-induced point (RIP) mutation in *Neurospora crassa* degrades transposable elements by targeting repeats with C→T mutations. Whether RIP affects core genomic sequence in important ways is unknown.

**Results:**

By parent-offspring whole genome sequencing, we estimate a mutation rate (3.38 ×  10^−6^ per bp per generation) that is two orders of magnitude higher than reported for any non-viral organism, with 93–98% of mutations being RIP-associated. RIP mutations are, however, relatively rare in coding sequence, in part because RIP preferentially attacks GC-poor long duplicates that interact in three dimensional space, while coding sequence duplicates are rare, GC-rich, short, and tend not to interact. Despite this, with over 5 coding sequence mutations per genome per generation, the mutational burden is an order of magnitude higher than the previously highest observed. Unexpectedly, the majority of these coding sequence mutations appear not to be the direct product of RIP being mostly in non-duplicate sequence and predominantly not C→T mutations. Nonetheless, RIP-deficient strains have over an order of magnitude fewer coding sequence mutations outside of duplicated domains than RIP-proficient strains.

**Conclusions:**

*Neurospora crassa* has the highest mutation rate and mutational burden of any non-viral life. While the high rate is largely due to the action of RIP, the mutational burden appears to be RIP-associated but not directly caused by RIP.

## Background

On the average mutations are deleterious [1]. As a consequence, classical theory predicts that selection will act to reduce the mutation rate [1]. In a few curious exceptional circumstances, however, organisms target sequence for mutation. Somatic hypermutation associated with immune system variation generation in mammals is one striking example [2]. Numerous fungi present a further unusual exemplar in the form of repeat-induced point mutation (RIP) [3]. RIP targets duplicated sequence and causes exceptionally high rates of C→T mutations within this duplicated sequence [4]. RIP occurs just immediately prior to meiosis after cell fusion but before nuclear fusion. While the process of meiotic homology searching might be an obvious system to redeploy to enable recognition of duplicate sequence, RIP does not involve the meiotic mechanisms [5]. The detection of duplicates in RIP is, for example, independent of homology searching associated with spo11/mei3 [6]. Rather, it is thought that RIP is mediated by the conserved pathway that establishes transcriptional (heterochromatic) silencing of repetitive DNA [3].

This mechanism is consistent with RIP having evolved as a genome-level defense mechanism against mobile elements [3] as, by definition, successful transposable elements (TEs) are those that can generate duplicate/repeat copies of themselves. RIP is commonly augmented by more classical TE suppression systems such as methylation [3]. Indeed, fungi have a rich suite of systems that counter TEs [3] including cotranscriptional RNA surveillance, meiotic silencing by unpaired DNA (MSUD), methylation induced premeiotically (MIP), sex-induced silencing (SIS), and cosuppression (alias somatic quelling).

The extent to which RIP is restricted to repetitive DNA and does not affect what may be considered the “core” genome has yet to be directly studied. This is a potentially important question as, were *Neurospora* to have both a high mutation rate and high mutational burden (i.e., deleterious mutations in coding sequence), then this would suggest that it trades off a mutational burden against the advantages of reduced TE load. This in turn would suggest an occurrence in which selection favors an increased mutation rate, even though many of these are deleterious. This would thus present a possible counter example to conventional wisdom regarding mutation rate evolution [1], namely that selection always favors reduced rates of heritable mutations.

There have been numerous single gene analyses of the mutation rate in *Neurospora* [7–12] that Lynch and colleagues [1] assembled to derive a figure of 4.10~4.66 × 10^−9^ point mutations per site (see Additional file 1: Supplementary Notes). At first sight, this estimate is comparable to that seen in, for example, *Plasmodium* (2.1 × 10^−9^) and *Trypanosomes* (1.4 × 10^−9^) [1]. However, the number is far from well resolved. Drake, for example, estimated the number of replications from the changes in population size and obtained 4.5 × 10^−11^ per bp per replication for *ad-3AB* and 1.0 × 10^−10^ per bp per replication for *mtr* [13], averaging at 2.3 × 10^−11^. Despite this heterogeneity, the rate appears be unexceptional. This suggests in turn that RIP does not affect the core genome. Such an inference is, however, premature. First, if the limited range of sequences analyzed do not include any that RIP would see as “duplicate,” they would fail to reflect what might be happening genome wide. Further, even if not duplicate, as RIP is known to leak into sequence flanking sites identified as duplicate [3, 14], if the single gene sequences analyzed were also not in proximity to sites that are RIPed, they have the potential to be unrepresentative. Possibly more importantly, some estimates were derived during asexual propagation (for discussion, see Additional file 1: Supplementary Notes), so missing any RIP component, RIP being pre-meiotic.

Estimation of the mutation rate in the face of these limitations calls for an unbiased whole genome sequencing (WGS) approach via parent-offspring sequencing. A subsidiary question is whether coding sequence is relatively untouched and hence whether there is not a substantial mutational burden. While a priori we expect RIP to evolve to cause little collateral damage, the extent to which RIP can mutagenize TEs and leave CDS untouched is unknown. If coding sequences and non-coding sequences are differently exposed to RIP, then attempting to partition causes of mutation through analysis of a single coding gene (e.g., [9]) is likely to provide biased estimates. In such circumstances, we need also to assay the mutational burden (the number mutations affecting CDS per genome per sexual generation [1]), by direct observation of mutations in CDS, rather than via extrapolation from the amount of the genome that is coding and the mean per base mutation rate [1]. Our WGS data allow us to provide an unbiased estimate of *Neurospora*’s mutational burden.

To address the impact of RIP, as opposed to other sources of mutation, we consider a variety of strategies. First, we examine asexual lines, employing them to estimate, by extrapolation, the expected number of mutations through the sexual cycle. This has the disadvantage that it requires us to assume that RIP is the only mutational process that is different between the asexual and sexual cycles which may well not be true [9]. To overcome this limitation, we also employ sexual strains deficient for RIP. One such strain we discovered by serendipity, strain FGSC2225, was found to be a natural *rid* mutant and hence naturally at least partially RIP deficient. We exclude its cross (C) from initial calculations. However, *rid* mutants may not be fully RIP deficient. As a further check then, we derived *rid* (−/−), *dim-2* (−/−) double mutants. In addition, we also consider the location and type of mutations observed in the RIP-proficient strains. RIP mutations are classically reported as C→T mutations [4] (note that for convenience, we refer to C→ T mutations, understanding that these can also be reported as G→A). We also expect them to be in “duplicate” domains and clustered within such domains. Four different approaches to define “duplicate” have been considered, one of which (a Blast-based approach) we find to be the most efficient in capturing both nearly all of the excess of mutation and having a high sequence to mutation rate ratio (see the “Methods” section). Unless otherwise stated, this is our operating definition of “duplicate”.

We estimate that *Neurospora* has a mutation rate per bp per sexual generation that is two orders of magnitude higher than any estimate for any cellular (i.e., non-viral) life form [1]. The great majority of these mutations appear to be RIP-associated. As perhaps expected, RIP seems to be structured in a manner that largely avoids coding sequence. It preferentially attacks GC-poor long duplicates, while CDS-associated duplicates are uncommon, GC-rich, and short. Despite this, we also discover an unexpected result: while the number of CDS mutations per genome per generation is one order of magnitude higher than the prior record (held by much larger genomes of humans, chimps, and rice), this is not owing to the direct impact of RIP. Indeed, the CDS mutations are for the most part not classical RIP type (i.e., not greatly skewed to C→T events) and not associated with duplicate domains, even with the most liberal definition of what might be duplicate. Nonetheless, the most RIP-deficient strain has over an order of magnitude fewer CDS mutations outside of duplicated domains. These results can be rationalized if RIP is also associated with a more general genome-wide increase in the mutation rate that is not of classical RIP form or location. We discuss implications of our results for mutation rate theory, timing of RIP, and theories of sex.

## Results

### *Neurospora* has an extreme mutation rate

**Fig. 1:**
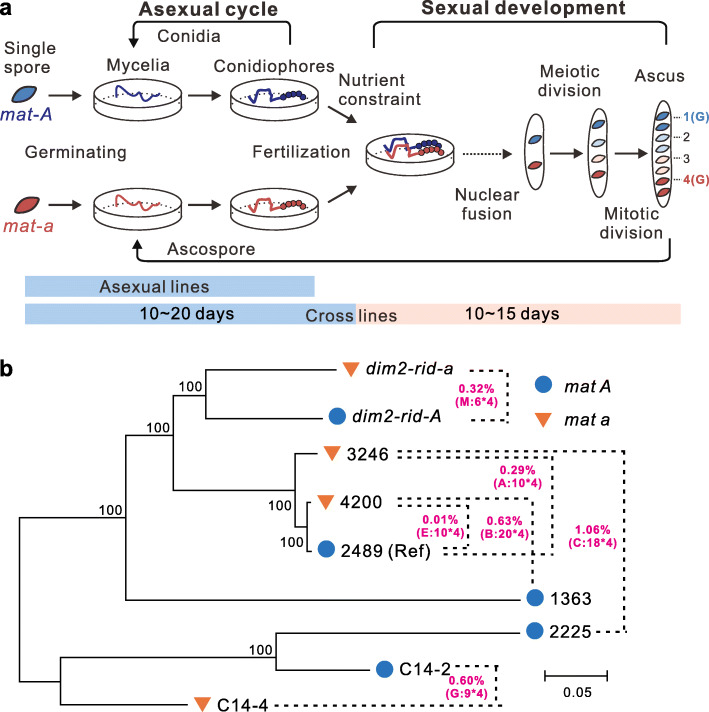
Preparation of materials for estimation of mutation rates in *Neurospora crassa*. **a** Experimental design. The asexual cycle includes the germination of vegetative spores (conidia), formation of mycelia, emergence of conidiophores, and further formation of conidia. The sexual cycle involves two spores (either asexual conidia or sexual ascospores) of opposite mating types, mating type *A* (blue) and mating type *a* (red). When subject to nitrogen starvation, either mating type can form “female” structures for the opposite mating type to fertilize and initiate development of perithecia. The nuclei of two mating types would coexist in the same cytoplasm and undergo mitoses before fusion. A meiotic division followed by a mitotic division would be initiated immediately after the fusion of two haploid nuclei, which generates the ascus with eight ascospores. Illustration based on Aramayo and Selker [15]. Dashed arrows: some intermediate steps are not shown. The approximate stages covered in each experiment are indicated below. For crossing lines, four non-mitotic sister spores (1~4) were sequenced. For cross group G, the two parents were selected from the products of cross group C (e.g., ascospore 1 and 4). **b** SNP phylogeny of parental strains used to generate the six crosses. Two parental strains for each cross are connected by dashed lines, with the estimated diversity given in proximity. The cross ID and numbers of sequenced asci (× 4 non-sister spores) are given in parentheses. The phylogenetic tree was constructed using SNP sites only (neighbor-joining method, 1000 bootstrap replicates)

We selected four non-sister ascospores from the eight products of meiosis to perform whole genome sequencing (Fig. 1a). A total of 273 *Neurospora* haploid samples, including 5 parental strains and 67 tetrads (67 × 4 = 268 samples) from five different crosses, were sequenced (see Materials and Methods, Fig. 1b and Additional file 2: Datasheet S1). The parental strains have an average SNP diversity of 0.52% (range 0.01 to 1.06%) and should thus largely avoid hybridogenic/heterozygous-induced mutation [16, 17]. Mutations were then identified through comparing each sequenced ascospore against parental strains.

Analytical artifacts are negligible owing to *N*. *crassa* being haploid (Materials and Methods, Additional file 2: Datasheets S2 and S3). From Sanger resequencing, we estimate a 0% false positive rate (see the “Methods” section). From simulation and resequencing, we estimate a less than 10% false negative effect (owing to parts of the genome with lower than necessary coverage—see the “Methods” section). Our estimates are normalized to the proportion of the genome covered (in any particular cross and in any particular class of domain) so allow for false negatives.

For RIP-proficient strains, we find that through the sexual cycle, there are on average 136.6 (± 21.5, sem) mutations per genome per generation (Table 1), equating to 3.38 × 10^−6^ (± 5.30 × 10^−7^ sem) per bp per generation. While this is three orders of magnitude higher than the 4.10~4.66 × 10^−9^ per bp rate previously estimated [1], that estimate is not per sexual generation so not directly comparable. To estimate the spontaneous mutational burden, we consider the number of CDS mutations per genome per generation. We observe on average 5.34 (± 0.46, sem) such mutations per genome per sexual generation (Table 2).

**Table 1:** Mutations over the sexual cycle of *N. crassa*. Number of mutations per genome per sexual cycle were reported for each genomic region, i.e., within duplicates (defined by Dup-Blast), near duplicates (400 bp upstream and downstream flanking regions) and non-duplicates. All numbers are given as mean ± SEM. 2:2 and 3:1 refer to the pattern of segregation of mutations post meiosis

Regions	2:2 mutations		3:1 mutations		Total	
	Average	Rate (× 10^**−8**^)	Average	Rate (× 10^**−8**^)	Average	Rate (× 10^**−8**^)
Within duplicates	119.2 ± 20.6	1810 ± 313	0.91 ± 0.22	13.8 ± 3.30	120.1 ± 20.7	1820 ± 314
Near duplicates	3.6 ± 0.5	129 ± 18	0.22 ± 0.08	7.92 ± 2.78	3.8 ± 0.5	137 ± 18.5
Non-duplicates	12.5 ± 1.1	40.2 ± 3.48	0.20 ± 0.09	0.632 ± 0.293	12.7 ± 1.1	40.9 ± 3.52
All	135.3 ± 21.4	334 ± 52.9	1.32 ± 0.30	3.27 ± 0.74	136.6 ± 21.5	338 ± 53.0

**Table 2:** Mutations within and outside of coding regions in *N. crassa*. For each genomic region, i.e., within duplicates (defined by Dup-Blast), near duplicates (400 bp upstream and downstream flanking regions), and non-duplicates, the mutations were counted within and without CDS. For cross lines, the numbers are given as “number of mutations per genome per sexual cycle.” For asexual lines, the numbers are given as “number of mutations per conidium per day for asexual cycle.” The C→T or G→A mutations within or near duplicates are given in square brackets

Regions	Regular crosses				Asexual lines		Cross C with FGSC2225 in origination		***dim2 rid*** double mutant cross	
	2:2 mutations		3:1 mutations							
	Coding	Non-coding	Coding	Non-coding	Coding	Non-coding	Coding	Non-coding	Coding	Non-coding
Within duplicates	0.231 [0.212]	119.0 [102.8]	0.02 [0.02]	0.89 [0.86]	0	0.201 [0.047]	0	1.08 [0.25]	0	1.49 [1.05]
Near duplicates	0.307 [0.140]	3.25 [1.75]	0	0.22 [0.21]	0.004 [0]	0.019 [0.004]	0.283 [0.283]	0.21 [0]	0	0
Non-duplicates	4.73 [1.49]	7.79 [3.11]	0.05 [0.04]	0.14 [0.12]	0.097 [0.029]	0.045 [0.016]	1.389 [0.404]	1.69 [0.93]	0.169 [0]	0.256 [0]
All	5.27 [1.84]	130.0 [107.7]	0.07 [0.06]	1.25 [1.19]	0.101 [0.029]	0.265 [0.067]	1.672 [0.687]	2.98 [1.18]	0.169 [0]	1.75 [1.05]

**Fig. 2:**
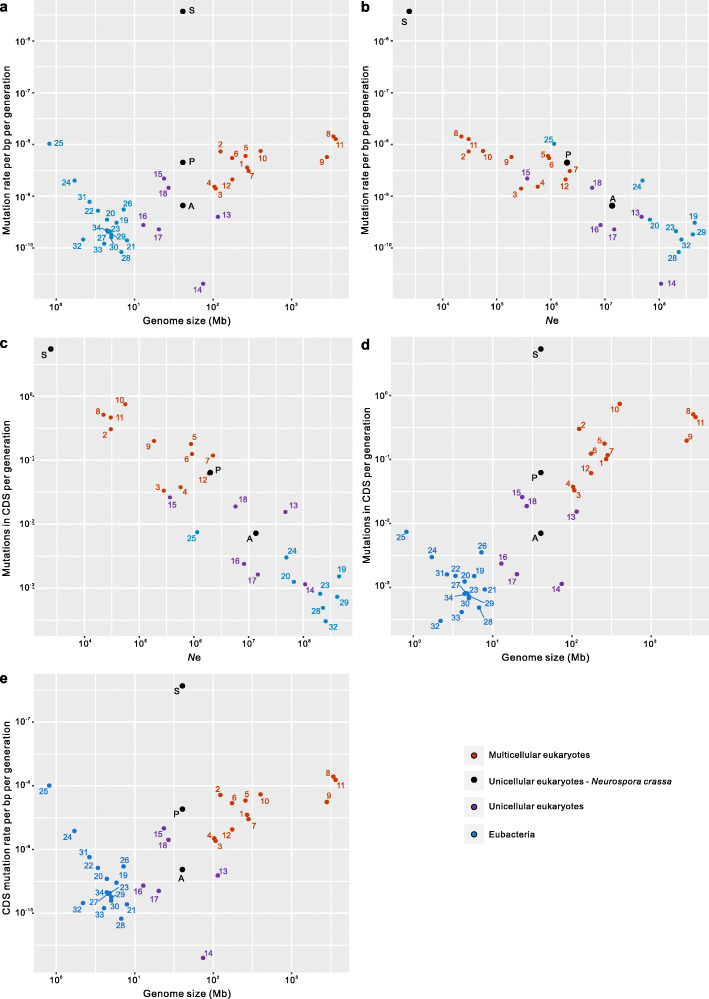
Relationships between predictor variables and mutation rate across taxa. **a** Genome size versus mutation rate per bp per generation. **b** Effective population size, *N*_e_, versus mutation rate per bp per generation. **c** Effective population size *N*_e_ versus number of mutations in CDS per genome per generation. **d** Genome size versus total number of mutations in CDS per genome per generation. **e** Genome size versus CDS mutation rate per bp of CDS per generation. Three *Neurospora* figures are presented in black dots: S is the rate/number over the sexual cycle as herein estimated; A is the rate per asexual division, herein estimated, and P is the rate as previously estimated [1]. For source data and species/number cross-referencing, see Additional file 1: Table S7

To consider these numbers in context, we compare them to those observed for other species. We consider five comparisons. First, genome size has been considered a predictor of the per base pair rate [13], at least for single-cell organisms [1]. As can be seen (Fig. 2a), the above rate is an outlier both compared to single-cell species and multicell species, being two orders of magnitude higher than any such prior estimates. With information on the level of diversity in intronic sequence or presumed neutral sequence [1], we estimate the effective population size, *N*_e_, considered the best predictor of the mutation rate [1]. *Neurospora* through the sexual cycle sits at an extreme end of the distribution in a plot of *N*_e_ against per bp rate (Fig. 2b) (though note that for unicells, the rates are per cell division; for multicells, they are typically per sexual generation). *Neurospora* is also an outlier in a plot of genomic CDS mutations predicted by *N*_e_ (Fig. 2c), its 5 CDS mutations per generation being an order of magnitude higher than the next highest estimates (for human, chimp, and rice). To provide a comparison controlling for genome size, one can see (Fig. 2d) that *Neurospora*’s number of CDS mutations is 2–3 orders of magnitude higher than genomes of comparable size. As this does not control for variation in the amount of CDS, we can also compare CDS mutations per bp of CDS (Fig. 2e). Again, we see a 2–3 orders of magnitude higher rate in *Neurospora*. As deleterious mutations can also occur in promoters, enhancers, and introns, and because most such mutations are deleterious, *Neurospora* has an exceptionally high load of spontaneous deleterious mutations.

### Most mutations are pre-meiotic in origin

We take several approaches to ask whether the mutations are likely RIP derived. First, we eliminate mutations that occurred during meiosis or in the single mitosis after meiosis (by which a tetrad becomes an octad). Premeiotic mutations will have a 2:2 segregation ratio while those post-RIP will have a 3:1 ratio. This is a minor correction as approximately 99% (135.3/136.6) of all mutations are by this definition premeiotic in origin (Table 1). 5.27 of the 5.34 CDS mutations per genome per generation are 2:2 segregating mutations (Table 2).

The correction is probably very conservative as many of the 3:1 mutations may well be late resolved RIP mutations. Indeed, similar to 2:2 mutations, higher mutation rates within or near duplicates were found for 3:1 mutations (Table 1). There was also a high proportion of C→T mutations among the 3:1 mutations (Additional file 1: Table S1), especially when related to duplicates. Around 52.8% of 3:1 mutations were found to be clustered C→T changes (the proportion is 78.9% within duplicates defined by Blast, 47.1% near duplicates, and 47.4% in non-duplicates). Clustering is defined as at least two mutations within 1 kb in any given spore (see also Materials and Methods). In RIP knockout lines, we found no 3:1 mutations. These results support the notion that meiotic mutations are rare in this species [9].

We now apply three approaches to ask whether the remaining 2:2 mutations are likely to be RIP derived. First, we ask about the expected number through an asexual cycle of the same length. Second, we consider the rates in the sexual cycle of RIP-deficient strains. Finally, we ask about the profile of the mutations in RIP proficient crosses. RIP mutations should be (a) C→T mutations that are (b) in or near duplicate domains that occur (c) in a clustered fashion [4].

### The asexual mutation rate suggests that over 90% of mutations are associated with the sexual cycle

Some of the 2:2 events likely reflect mitotic mutations that occurred prior to RIP. We determine the rate through mitosis per day and calculate the expected rate across the cycle in the absence of RIP. Two independent asexual lines, each grown starting from exactly a single spore, were propagated for 32 and 36 days respectively (asexual cycle in Fig. 1a). For each line, mycelia samples were collected every four days during propagation. A total of 81 samples, including 2 starting samples and 79 propagated samples, were sequenced (Additional file 2: Datasheet S1). From these, we estimated a mutation rate of 9.05 × 10^−9^ per site per day or roughly 6.03 × 10^−10^ per site per cell division (assuming 15 divisions per day [18]) during asexual propagation (Additional file 2: Datasheet S2). Even generously assuming 20 days for the sexual cycle in the lab, we estimate a net rate of 1.81 × 10^−7^ over the cycle. This is an order of magnitude lower than the observed rate seen in 2:2 segregants (3.34 × 10^−6^ per bp per generation), suggesting that at least 93% (=[3.34–0.181]/3.38) of all new mutations are RIP/sex associated (note that prior evidence suggests sources of non-RIP derived mutations that are particular to the sexual cycle [9]).

Similarly, we can estimate the expected number of mutations that affect CDS. We find 0.1 CDS mutations per genome per day (0.0067 per cell generation) hence [5.27–0.1 × 20]/5.34 = 61% of all CDS mutations are 2:2 mutations (likely RIP-associated) specific to the sexual cycle (Table 2). This number equates to a mutation rate of 6.79 × 10^−9^ per coding site per day or 4.52 × 10^−10^ per coding site per cell generation during asexual propagation. The figure of 0.0067 is comparable to what is seen in other fungi [1], notably *Saccharomyces cerevisiae* (0.0023) and *Schizosaccharomyces pombe* (0.0016), especially when adjusting for differences in total CDS in the genomes (*S. cerevisiae* would have 0.0038 were it to have the same volume of CDS and *S. pombe* would have 0.0032). The discrepancy between our estimate and the prior aggregate estimate of 0.06 [1] is possibly indicative of the complex mix of experimental approaches, some solely asexual, some sexual, that contribute to the prior aggregate. A lack of clarity of the units may also be contributory, it not being clear if the figure is a per mitosis rate. The prior estimate was also made under the assumption of a uniform mutation rate, such that the CDS rate can be estimated as the genomic mutation rate scaled to the proportion of the genome that is CDS. With RIP’s concentration on repeat elements, an assumption of uniformity is probably invalid.

### Analysis of RIP-deficient strains suggest that over 95% of mutations are RIP derived

The above estimates require assumption about the similarity of asexual and sexual cycles and require assumptions as to how to extrapolate from one to the other. To address these limitations, we consider RIP-deficient strains through the sexual cycle.

First, we considered cross C involving the *rid* deficient strain FGSC2225. Nearly all mutations were from the other parental strain (Additional file 1: Table S2). There are 84 mutations (after false negative rate control) found with FGSC2225 in origination (Additional file 2: Datasheet S2). From this, we could estimate a RIP-deficient rate of 84/18 per asci = 4.67 per ascus. This in turn translates to a rate of 1.15 × 10^−7^ per site per sexual generation, assuming a reference genome size of 40.46 MB. In turn, this implies that ([135.3–4.67]/136.6) = ~ 95% of mutations are RIP-associated.

As the above mutant may have residual RIP activity, in addition, we derived a *rid* (−/−), *dim-2* (−/−) double mutant cross (Fig. 1b). A total of 13 mutation sites were detected among 6 sequenced asci (Additional file 2: Datasheet S2), yielding 1.92 normalized mutations per genome per cycle, a per site per sexual cycle rate of ~ 4.7 × 10^−8^, about over 50-fold lower than the 3.38 × 10^−6^ per site per sexual cycle rate estimated for the RIP proficient normal crosses (Table 1). This cross indicates ([135.3–1.92]/136.6) = 98% of mutations are RIP derived and indeed suggests that the double mutation ablates RIP slightly more than the single *rid* mutant.

From the above, we estimate that between 93% (from asexual extrapolation) and 98% (from the double RIP mutant) of new mutations are likely associated with RIP, with the single RIP mutant agreeing with this span. We are hesitant to extract too much from small discrepancy between the asexual estimate and the double knockout estimate, not least because this could be owing to error in extrapolation (e.g., employing the wrong number of days). If 98% of mutations in the sexual cycle are RIP-associated, this implies a very minor role for mutations that are associated with the sexual cycle but that are not RIP-associated [9].

### The majority of CDS mutations are RIP-associated

We can use the same analysis as presented above to estimate the number of CDS mutations in RIP-deficient lines. We find 1.67 (with 0 in duplicates and 0.28 C→T mutations near duplicates) and 0.169 (with no mutations in and near duplicates) CDS mutations per genome per cycle with FGSC2225 in origination and from the double mutant cross, respectively (Table 2). As FGSC2225 likely had some residual RIP activity having an intact *dim-2* locus, we suggest that the latter figure is the better estimator. This compares to 5.27 2:2 CDS mutations in normal crosses. Assuming the difference reflects RIP activity, we estimate that between 67.4% and 95.5% of CDS mutations are 2:2 mutations in the sexual cycle that are RIP-associated, the latter being probably the more reliable estimate. The asexual lines have about 0.1 × 20 = 2 CDS mutations in 20 days, similar to that seen for the *rid* mutant and suggesting that 61% of CDS mutations are RIP-associated.

### Most mutations are classical RIP C→T mutations clustered on the same strand in duplicated domains

**Fig. 3:**
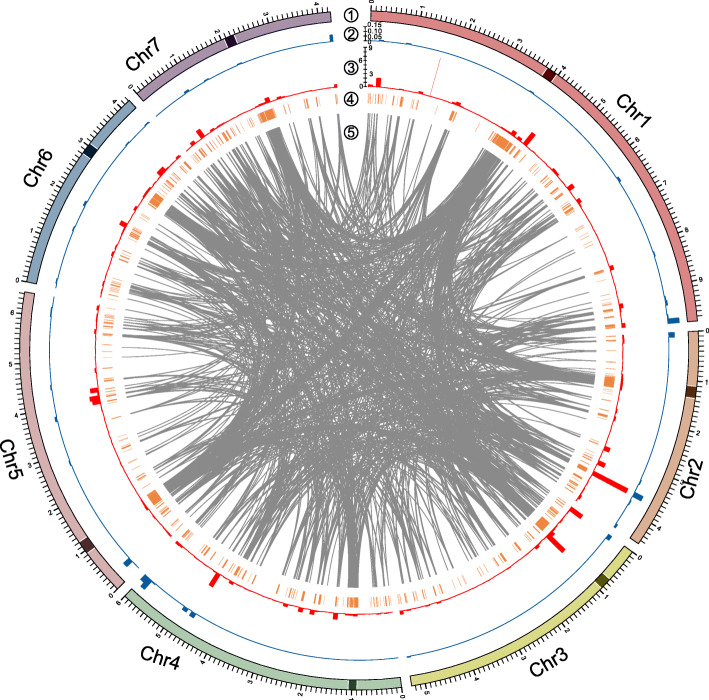
Genome-wide distributions of duplicates and mutations. Tracks from outer to inner: ① seven chromosomes, black box represents putative centromeric regions; ② sexual 3:1 mutations per 100 kb, blue circle, scale bar represent 0~0.15; ③ sexual 2:2 mutations per 100 kb, red circle, scale bar represents 0~9; ④ duplicate regions (Dup-Blast); ⑤ best matched duplicates were linked by gray lines. Only 2000 randomly picked best hits with alignable length over 150 bp and identity over 85% are shown here for better visualization

If owing to RIP, we expect the majority of mutations in normal sexual lines to be C→T mutations clustered in duplicate domains (Additional file 1: Supplementary Notes, Figure S1 and Table S1). Naturally, this tempts the question as to what *Neurospora* considers to be “duplicate”. We consider four different definitions (see the “Methods” section) and find that the most efficient method to define duplicates is via Blast (see the “Methods” section). Efficient in this context implies both a high ratio of mutations to the proportion of sequence defined as duplicate and an absolutely high proportion of the mutational excess seen in comparison of RIP proficient and RIP-deficient lines. Using a criterion of over 65% identity [19] and at least 100 bp of alignable sequence [6] to define “duplicate” sequence (denoted as the Dup-Blast method), around 16% of the reference genome belongs to such sequences (Additional file 1: Figure S2, Table S3, and Additional file 2: Datasheet S4). Approximately 87.4% and 2.9% of detected 2:2 mitotic mutations were found within or around (400 bp upstream and downstream) those regions respectively (Table 1). These numbers correspond to a per site rate of 1.81 × 10^−5^ ± 3.13 × 10^−6^ sem and 1.29 × 10^−6^ ± 1.80 × 10^−7^ sem, in duplicate and duplicate-proximal sequence respectively. The per site rates are 40- and 3- fold higher than in the non-duplicate sequence (4.02 × 10^−7^ ± 3.48 × 10^−8^ sem), respectively (chi-squared with Yates’ correction, Dup vs non-Dup, *χ*^2^ = 71,684, *P* < 2.2 × 10^−16^; Dup-proximal vs non-Dup, *χ*^2^ = 426, *P* < 2.2 × 10^−16^). For the location of duplicate sequence, see Fig. 3.

The profile of mutations further implicates RIP as the dominant source of mutation. We found 91.8% of 2:2 mitotic mutation events in duplicates were classical RIP-associated C→T base changes (Additional file 1: Figure S1 and Table S1). If the BLAST method underestimates the sequence that is duplicate [20], this provides a lower bound estimate. The proportion of C→T changes was also higher in duplicate-proximal regions compared to non-duplicate domains, which were 66.7% and 42.7%, respectively (chi-squared with Yates’ correction, *χ*^2^ = 38.8, *P* = 4.57 × 10^−10^). These results are congruent with our estimates (from asexuals and knockouts) that about 95% of mutations through the sexual cycle are RIP-associated.

As expected of RIP, C→T mutations in duplicate domains were usually found to be clustered within a small genomic range. Most 2:2 mutations (~ 76.3%) tightly clustered within the mutated duplicates (Additional file 1: Figure S3 and S4, Table S4 and Additional file 2: Datasheet S5), of which around 99.4% were C→T changes (Additional file 2: Datasheet S5). The mean distance to the nearest mutation genome-wide is ~ 6.3 kb but the median is just 41 bp as over 60% of mutations are found within only 100 bp of the nearest neighbor (Additional file 1: Figure S4).

The mutations were also “strand-biased” [21, 22], with approximately 91.9% of the mutation clusters containing either exclusively “C→T” or exclusively “G→A” changes, but not both, within a single strand (Additional file 1: Figure S4 and Additional file 2: Datasheet S5). This strongly supports the idea that one round of RIP acts on one strand at a time [22]. The remaining 10% of mutation clusters were also dominated by C→T changes, with only 64 non-C→T mutations (Additional file 2: Datasheet S5). Of the 64 non-C→T clustered mutations, 6 were present within the C→T clusters, while others form small clusters which have only two mutations for each cluster.

### Mutations through the asexual cycle have a different profile to those through the sexual/RIP cycle

While the bulk of mutations have a RIP profile to be confident that they are RIP derived, we also need to consider the negative control, mitotic mutations. Importantly, the profile of RIP mutations is different from that of mitotic changes. The identified mutations during the pure asexual cycle are much less skewed in their spectra with only 23.4% C→T mutations (Additional file 1: Figure S1, Table S1 and Additional file 2: Datasheet S2). They were, however, still enriched, albeit to a lesser extent (just 4-fold), in duplicates (44.4% within Dup-Blast duplicates, expected = 16.3% within duplicates, *χ*^2^ with Yates’ correction = 52.4, *P* = 4.53 × 10^−13^; and 6.7% around duplicates, expected = 6.8% around duplicates, *χ*^2^ = 0.003, *P* = 0.954) compared to those from the sexual cycle (Additional file 2: Datasheet S2). This skew towards duplicate sequence goes at least some way to explain why in the sexuals we observe 9.5% of mutations within duplicates that are not C→T changes and only rarely present in clusters.

Many (51.5%) of the asexual mutations within duplicates are in centromeric duplicate sequence (which is 22.8% of duplicates), while only 10.5% of the sexual mutations within duplicates are centromeric (Additional file 1: Table S5 and Additional file 2: Datasheet S2). Only rarely were clustered mutation hotspots seen in the asexual cycle which contrasts to the extensive presence of mutation clusters from the sexual cycle.

### The double knockout cross is largely (but possibly not entirely) devoid of RIP activity

If we take the profile of mutations through the asexual cycle as a null, we can ask whether the double knockout shows evidence of residual RIP activity, based on the class of mutations and their clustering. Analysis of the form and location of the mutations suggest that the double knockout cross is largely, but possibly not entirely, devoid of RIP activity.

Of the 13 mutation sites, only 5 are RIP-type C→T changes; the remaining 8 are non-RIP C→T changes (Additional file 2: Datasheet S2). Assuming that the asexual lines provide a fair comparator, we would expect 3.042 C→T and 9.958 non C→T, which is not significantly different from what is observed (with expectation drawn from asexual proportions, *χ*^2^ with Yate’s correction = 0.91, df = 1, *P* > 0.05). This suggests little or no residual RIP activity. Overall, 9 of 13 (69.2%) of mutation sites were found in repeat regions, compared with 44.4% in asexuals (Additional file 2: Datasheet S2) (with expectation drawn from asexual proportions, *χ*^2^ with Yate’s correction = 2.41, df = 1, *P* > 0.05). However, this disguises the observation that all the C→T changes are in repeat regions while, of the eight others, four are in repeat regions. Most suggestively, four of the “C→T” mutations sites were in a cluster, this being the only mutation cluster found in the mutant cross. This is indicative of a small residual degree of RIP even in the double knockout, worthy of further scrutiny.

### Coding sequences are not commonly RIPed

The above at first sight implies that the majority, probably the overwhelming majority, of mutations and CDS mutations are associated with RIP. However, closer scrutiny of the mutations that affect CDS and non-duplicate sequence suggests both that RIP is especially rare in CDS but that, nonetheless, RIP is associated with a generally raised mutation rate at non-classical RIP sites.

The observed > 5 CDS mutations per genome is less than expected under a random null given that CDS is 37.5% of the genome (expected ~ 50 CDS mutations per genome per generation). At first sight, this can in part be accounted for by the rarity of CDS in duplicate, with only 2.2% of CDS within “duplicate” domain. We also find that not all duplicates are affected equally, with longer duplicates, GC poor ones and those with more common 3D connections having higher RIP rates (Additional file 1: Supplementary Notes, Figure S5). CDS duplicates appear to have properties that suggest that even if they are in “duplicate,” they are not in the most RIP prone class of duplicate sequence. CDS “duplicate” sequence tends to be both shorter (338.1 bp ± 24.04 bp sem v 2029.9 bp ± 85.25 bp, sem) and GC richer (54.6% ± 0.17%, sem v 37.4% ± 0.19%, sem) than non-CDS duplicate. In addition, among all 2376 non-ambiguous windows with putative Hi-C interactions, 1920 windows overlap with CDS and 897 windows overlap with duplicates. Most non-CDS duplicates could have interactions (443 of 456 non-CDS interacted windows are duplicates = 97.1%), but fewer CDS duplicates have interactions (454 of 1920 CDS interacting windows are duplicates = 23.6%: chi-squared test with Yates’ correction = 844.04, *P* < 2.2e−16).

These results suggest that CDS sequence should be protected from RIP by being GC rich, short and not interactive in 3D. Indeed, while the sexual cycle is associated with RIP in duplicates, very few such duplication-associated mutations are in CDS (12 v 6766 2:2 mutations = 0.177%: Table 2). Similarly, of the 296 2:2 CDS mutations in our sample, only 7.1% (*N* = 21) are classical RIP-associated C→T in or near duplicate sequence (Additional file 2: Datasheet S2). While we cannot ever be sure that the definition of “duplicate” captures all domains that are duplicate in the eyes of RIP, the evidence is strongly consistent with a rarity of RIP-induced mutation in CDS.

### RIP is associated with a raised mutation rate outside of classical RIP zones

That RIP largely avoids CDS is to be expected in selectionist terms given how mutagenic the process is. One can imagine a system tolerating a high mutation rate within transposable elements (old or new) that leaves CDS intact. It is then unexpected that the rate of such CDS non-duplicate mutations through the sexual cycle is much higher than seen in RIP-deficient lines. Even with the most conservative non-duplicate definition (i.e., non-duplicate by any of four definitions, see Materials and Methods), the CDS mutation rate is 3.62 per genome per cycle for sexual 2:2 mutations in non-duplicate sequence, only ~ 30% of the 3.62 mutations being C→T mutations (Additional file 1: Table S6). This frequency is comparable to that seen in asexual lines (23%) and lower than that in the RIP double knockout (38%) indicative of not being directly affected by RIP. This non-classical RIP rate in CDS is 40 times higher than in RIP double mutant cross, which is 0.085 per genome per cycle in the same non-duplicate sequence (Additional file 1: Table S6). Analysis of the RIP-deficient natural strain supports the same raised RIP-associated, but non-classical RIP, rate in non-duplicate domains, but suggests that this effect may be more modest. In the same non-duplicate sequence, FGSC2225 has 0.985 CDS mutations per genome (Additional file 1: Table S6). While an order of magnitude higher than in the fully RIP-deficient strain, it is still 3.6 times lower than in the normal strains.

RIP coupled with the sexual cycle appears then to be associated with a massively increased mutation rate in sequence in or near repeats (classical RIP), but is also, unexpectedly, associated with a raised mutation rate outside of classical RIP domains, these mutations being different from classical RIP mutations. These RIP-associated, non-classical RIP mutations account for the majority of CDS mutations through the sexual cycle.

## Discussion

It is logically possible that RIP acts on duplicate sequence such that, after any new TE invasion has been quelled, RIP ceases to operate as there is no more “duplicate” on which to act. Here, we have shown that in numerous *Neurospora* strains, RIP is active in the absence of artificially introduced duplicate sequence (the usual mode of studying RIP). Moreover, the net mutation rate (per bp per generation) is orders of magnitude higher than anything previously reported for cellular life forms. Our whole genome estimate that ~ 95% of all mutations are RIP-associated contrasts with prior much lower estimates based on analysis of a single protein coding gene [9], in no small part because CDS and non-CDS sequence are differently exposed to RIP. That 98% can be attributed to RIP through a comparison of the double knockout and wild types strongly indicates that the order of magnitude differences that we observe between *Neurospora* and comparable species (Fig. 2) cannot be accounted for by, for example, variation in mutations in repair enzymes or modest differences in mutation rates between early and late sampled spores (and our protocol is not obviously biased one way or the other).

While RIP appears to largely avoid CDS, nonetheless strains with RIP have very high CDS mutation rates as well. As the CDS mutation rate in non-duplicate sequence in RIP-deficient lines is between 1/3 and 1/40 that of those with RIP, we suggest that there are both direct RIP-induced mutations (C→T, clustered in duplicates) and indirectly non-classical RIP-associated mutations (not necessarily C→T, not necessarily in duplicate and not clustered). The former accounts for the vast bulk of all point mutations; the later accounts for the majority of CDS mutations.

While our results are supported by analysis from asexual extrapolation, two RIP-deficient strains and by the profile of mutations, our observations come with the caveat that we are unable to fully resolve variation that might be owing to intrinsic strain differences (rather than, for example, being owing to knockout status). As we employed numerous different strains, our results are both likely to be representative of what is seen in the wild and not distorted by reliance on only one strain. We indeed observe high variation between RIP-proficient strains, even between replicates of the same cross (Table 1, Additional file 2: Datasheet S2). Similarly, with the strains we employ there appears to be a difference in mutation rates between mating types (Additional file 1: Table S2), but whether this reflects true differences between mating types or differences between the strains that happen to constitute our mating types we cannot resolve. This caveat is most important in the context of the RIP-associated CDS mutations that appear not to be the direct product of RIP. Our core evidence here is that that RIP double knockout has 1/40 the CDS mutations outside of duplicate sequence than do the RIP-proficient strains. However, both the single gene knockout strain and our extrapolation from asexuality suggest the effect to be rather weaker, more like 2/5–1/3 the rate. We consider the double knockout to be the best indicator as it appears to best mimic the sexual cycle in the absence of RIP, and even then RIP may not be fully abolished. The asexual estimates for example cannot factor in mutational events associated with the sexual cycle that are not RIP-associated [9]. Nonetheless, an analysis that could simultaneously control for strain effects and RIP effects would be desirable. A large variation between crosses, even when the crosses are between the same strains, suggests that this is not going to be simple.

### The mutation rate and deleterious mutational load are predicted by *N*_e_

Beyond an exemplar of a species with an extraordinarily high mutational rate and load, what are we to make of this circumstance? Classical mutation rate evolution theory assumes that mutations are mostly deleterious and thus that selection is favored to reduce mutation rates [1, 23]. One model suggests that the strength of selection to reduce the mutation rate is higher the greater the absolute number of functional sites in the genome [13]. A possible negative correlation between genome size parameters and the per base pair per cell division mutation rate was considered consistent with this model [13]. However, with the accumulation of more data even for single species, the validity is unclear and any correlation dependent on a few extreme values [1]. Our estimate for the per mitosis rate at 6.03 × 10^−10^ per site per cell division supports the view that the mitotic rate is not a monotonically decreasing function of genome size (Fig. 2a), even though this is an order of magnitude lower than estimates for genomes of comparable size.

An alternative model [24] suggests that, as selection on modifiers of the mutation rate is weak, the effective population size (*N*_e_) places a barrier on monotonic selection favoring reduced mutation rates. As effective population size is reduced, so the drift barrier is raised and the mutation rate per bp increases. One might suggest from Fig. 2b and c that, given their value of *N*_e_, *Neurospora* even in the per sexual generation calculation has (approximately) the mutation rate expected from the drift-balance model. That is to say, in both Fig. 2b and c*Neurospora*’s exceptionally high rates appear to sit close to a projected line (although this is less compelling in Fig. 2b). At first sight, this provides prima facie support for the drift barrier model.

This conclusion comes however with a potentially important caveat. As noticed [24], the existence of a negative correlation (as in Fig. 2b), and data sitting close to a line with negative slope, is not necessarily itself good evidence in favor of the model. Most notably, as the mutation rate estimation is imported to the calculation of *N*_e_, the null expectation need not be a flat line. The total number of CDS mutations versus *N*_e_ plot (Fig. 2c) is similarly affected as the CDS mutation rate is the proportion of the genome that is CDS multiplied by the genomic mutation rate (except for our *Neurospora* data where the number is generated directly as the assumption of uniformity of the mutation rate is severely abused). To estimate *N*_e_, Lynch and colleagues use a rearrangement of the classical formula relating polymorphism (*π*) to mutation rate (*μ*) and *N*_e_, such that *N*_e_ is then estimated by mutation rate and polymorphism:

*N*_e_ = *π*/*μ D*(*1* − *π*),

where *D* is a modifier reflecting either diploidy (*D* = 4) or haploidy (*D* = 2). Notably, as this formula presents *N*_e_ as a function of the inverse of the mutation rate [24], a negative correlation between the mutation rate and *N*_e_, or between *N*_e_ and the total number of CDS mutations is likely under several circumstances [24], even if the drift barrier model is wrong. Indeed, in Fig. 2b, we employ three very different estimates of *Neurospora*’s “mutation rate” but all appear to located approximately as expected given “underlying” *N*_e_. This, however, is owing to the fact that we employ the same estimate of polymorphism, giving three different predictions of “*N*_e_” for the same species, these three by necessity being negatively correlated with the three mutation rate estimates. Similarly, for example, if we were to consider that all values of polymorphism (*π*) are randomly drawn from the same underlying distribution, we then expect to recover a negative correlation simply owing to the methodology. Indeed, if we randomize diversity (*π*) figures between taxa and recalculate “*N*_e_,” then in all randomizations, we recover a negative correlation between “*N*_e_” and the mutation rate per bp per generation and between “*N*_e_” and number of CDS mutations per genome per generation, with nearly all (9996 of 10,000 simulations) having a significantly negative correlation (at *P* < 0.05). This is, however, a rather extreme null model and it seems unlikely that parameter estimation is this poor (and that polymorphism values reflect random samples from the same underlying distribution). Indeed, we find that, in the above simulations, the observed slope is one of the most negative compared with the randomizations (*P* = 0.027 for Fig. 2b, *P* = 0.0039 for Fig. 2c). We conclude that the fact that *Neurospora* in the sexual cycle sits near the projected line in a plot (Fig. 2b, c) is consistent with but, not decisive of, the relevance of *N*_e_ as a predictor.

Given the possibility that the negative correlation is potentially tautologous, we suggest that for evaluation of the drift barrier model, a method to infer *N*_e_, or proxies of *N*_e_, that does not require the implicit circularity would be helpful. Consideration of the extent of constraint on coding sequence (thought to be weaker when *N*_e_ is low) might make an alternative metric. This is left to future analysis.

### A trade-off between transposable element destruction and collateral damage?

Given that we identified natural variants that are deficient in RIP, *Neurospora* provides an unusual (if not unique) example of a sexual species in which a modifier (RIP proficiency) that increases the mutation rate persists despite it being associated with a raised mutational burden. Why then might *Neurospora* maintain a system that acts in the opposite direction? The most obvious possibility is that RIP is maintained as an anti-TE device. If so, our results suggest that mutation rate evolution is not always associated with selection for the minimal mutational burden and the minimal rate. Rather, advantages associated with targeting TEs can out way the burden of collateral damage.

Under such a model, we do not expect a unique stable mutation rate. Rather, the optimal rate at any given time would be dependent on TE burden and collateral mutational burden, but as RIP affects the TE burden, the optimum would be constantly shifting. The discovery of the natural RIP-deficient strain is consistent with such a lack of a stable optimal mutation rate. That RIP has been lost in some of *Neurospora*’s relatives (e.g., *Sordaria* [25]) and that we see large between-strain variance in RIP-associated mutation rates would similarly be consistent.

### RIP and theories of sex

That RIP acts within haploid nuclei is easy to rationalize. Were RIP to act in diploid cells (post nuclear fusion), it would potentially recognize all homologous sequence as duplicate, i.e., between homologous chromosomes. This would render all of the genome potentially subject to RIP. As it is, duplicate sequence must be “duplicate” within any given haploid genome. Why then is RIP-associated with cell fusion immediately before meiosis, rather than, for example, the haploid post-meiotic cells? We suggest that as sex and recombination can aid the purging of deleterious mutations [26, 27], and because RIP is associated with a raised burden, RIP’s timing is such that it enables immediate variation generation, thereby potentially generating cells with a low burden in spite of RIP. RIP after meiosis would result in all cells having a high burden. In short, by generating so many mutations and immediately recombining them, the system enables rapid removal of highly burdened lineages and survival of lineages with a lower burden than would be possible for any cell were RIP to be post-meiotic.

In turn, however, our results also suggest that models for the evolution of sex that evoke mutational purging [26, 28] make invalid assumptions for this species. These models assume that the sexual and asexual cycles that are in competition would have the same mutation rate and the same mutational burden. While the deleterious mutation rate in RIP-proficient sexual *Neurospora* is high enough to sustain sex according to the mutational deterministic model [26, 27], it having > 1 deleterious mutation per genome per generation (although this condition is particular to anisogamous species), asexual competitors do not have the same mutation rate, thus invalidating the model’s assumptions. At what point selection would favor a sexual strategy if this was associated with a higher burden of spontaneous mutations remains to be explored.

## Conclusions

*Neurospora* has the highest mutation rate and mutational burden of any non-viral life. While the high rate is largely owing to the action of RIP, the mutational burden appears to be RIP-associated but not directly caused by RIP. *Neurospora* presents an unusual exception to the rule in favoring a high mutation rate despite this being associated with a high mutational burden.

## Methods

### Source and description of parental strains

FGSC4200 and FGSC2489 were a gift from Tian Chaoguang, Tianjin Institute of Industrial Biotechnology, Chinese Academy of Sciences, China. FGSC2225 was a gift from Li Shaojie, State Key Laboratory of Mycology, Institute of Microbiology, Chinese Academy of Sciences, China. FGSC3246 and FGSC1363 were purchased from Fungal Genetics Stock Center, Department of Plant Pathology, Kansas State University, USA.

Both FGSC 2489 and 4200 are highly inbred strains and are preferred for use as standard wild types [29]. FGSC 4200 is a progeny from the 6th backcross to FGSC 2489 (the reference genome strain) of FGSC 2490. FGSC 2225 (Mauriceville-1cA, collected from Mauriceville, TX, USA) is a strain isolated from nature [30]. Strain FGSC 1363 is a morphological mutant that begins growth on agar as small colonies and sooner or later produces a flare of wild-type-appearing hyphae (with or without conidia) [31]. FGSC 3246 is a female sterile mutant [32]. From the SNP profile, there is no reason to suppose that they are hyper-mutators [33]. Prior examination of RIP in nature only identified very few wild isolates that dominantly suppress RIP [34, 35], suggesting similar properties of these lab strains in triggering RIP as in nature.

### Cross and ascospore dissection

All the crosses were mated and incubated under lab conditions. To avoid contamination, all culture media, as well as plates, were autoclaved at ~ 121 °C for over 25 min in high-pressure steam sterilizer (TOMY SX-500) before each experiment. Subsequent procedures were conducted on clean benches which had been sterilized using ultraviolet immediately prior to the experiment. The culture medium and experimental methods used in this research were based on the protocols offered by Fungal Genetics Stock Center (http://www.fgsc.net/). In brief, mycelium or ascospores of two mating strains were plated at the opposite end of the mating plate. If an ascospore was used as a mating parent, the spore was heat shocked at 60 °C, 30 min prior to plating. The crossing plates were incubated at 25 °C in a totally dark environment. After about 3 weeks of growth and crossing, the asci were separated and picked onto storage medium for a week. One ascus was picked on agar medium and incubated at 60 °C, 30 min. Then, the ascus was dissected under a microscope. Each ascospore in it was picked one by one onto its own growth medium. For cross G, two ascospores with opposite mating types from cross C were isolated and placed on the same crossing plate, which had undergone a round of sexual reproduction (Fig. 1a). The two spores for asexual propagation were originated from two ascospores from the cross of FGSC2225 and FGSC4200.

### DNA extraction and whole genome resequencing

Each of the ascospores dissected was cultured individually on its own plate for about 3 days so as to enable 3 micrograms or more of DNA to be extracted. DNA was extracted by phenol/chloroform/isoamyl alcohol method [36]. The DNA sample of each culture was extracted and re-sequenced individually.

Whole genome resequencing was carried out at Novogene (www.novogene.com) with the same protocol for all, involving 2 × 150 bp paired-end reads constructed by Illumina HiSeq 4000 platform. Two hundred seventy-three *N*. *crassa* samples (including 5 parental strains and 67 tetrads) were sequenced in total. On average, each spore was sequenced to a depth of ~ 37-fold with 96% of the genome covered. The parental strains were sequenced to a depth of ~ 76-fold with ~ 97% of the reference genome covered (Additional file 2: Datasheet S1). Over 92% of the reference genome could be covered by at least five reads in each sequenced sample, with a mis-mapping rate lower than 1% (Additional file 2: Datasheet S1).

### Identification of mutations

For consistency with prior estimates of the mutation rate (in different species), and because indel calling is prone to analysis artifacts, we restrict analysis to point mutations. The resequencing reads were mapped to the NC12 reference genome (https://www.broadinstitute.org/fungal-genome-initiative/neurospora-crassa-genome-project) using BWA aligner [37]. Variants were called by HaplotypeCaller from Genome Analysis Toolkit (GATK) [38]. Raw variants were filtered by removing any that were not called as “homozygous” or with a quality score less than 30. Given the haploid nature of samples, new mutations should all be called homozygous.

To be considered as candidate mutations we required further filters. The variant sites in offspring ascospores needed to be (i) different from their parents and (ii) have a supporting read-depth ≥ 5 both in the source parent and focal ascospores. Each candidate mutation was subsequently manually inspected to remove ambiguous results, including (1) sequencing errors, especially in polymer regions; (2) variants that actually existed in parent samples but failed to be captured by variant callers; and (3) artifacts from spurious alignments.

Though we were especially cautious during every step of the experiments, we cannot be 100% sure that no contamination was present in the sequenced DNA. However, we note that contamination should not confound our mutation calling for several reasons. First, if they have a chance to be mapped to the reference genome contaminants will generally give false “heterozygous variant calls,” but all the mutations that we employ are homozygous. “Homozygous calls” from contamination would only happen in the rare incidence in which the contaminant could be mapped to regions with large deletions in the sequenced strain (i.e., the contaminant is more similar to the reference genome than the sequenced strain). Second, contaminants usually give spurious alignments with clipped read ends as they tend not to map well to the reference genome, such alignments being discarded during mutation calling. Third, contaminants are expected to be randomly distributed among all collected samples, but mutations are either 2:2 or 3:1 segregated and generally present within a single ascus.

We estimate the false positive rate by Sanger resequencing. Of 186 mutations randomly picked for Sanger verification (Additional file 1: Figure S6 and Additional file 2: Datasheet S3), 14 could not be Sanger sequenced: three had noisy Sanger sequencing while 11 amplified the wrong sequence. In the 172 successful Sanger sequencings, all mutations were verified. We presume therefore a zero false-positive rate.

False negatives (FN) were estimated using two approaches. The first approach uses a simulation method similar to that described previously [39]. The empirical read-depth distribution was sampled from the real mutations identified. For each sexual cross, 5000 synthetic 2:2 mutation sites and 1000 synthetic 3:1 mutation sites were generated. For each asexual line, 1000 synthetic mutation sites were generated. The synthetic mutations were detected by the same pipelines, and the proportion of mutations correctly identified (1-false negative rates (FNR)) was calculated as “synthetic mutations identified”/“synthetic mutations generated” for each ascus. Since duplicates are more prone to mapping errors than non-duplicates, this FNR was calculated separately for three regions (denoted as regional FNR hereafter), i.e., within duplicates (defined by Dup-Blast), near duplicates (400 bp upstream and downstream of duplicates), and non-duplicates (Additional file 2: Datasheet S6).

The second approach is based on the Sanger sequencing results (Additional file 2: Datasheet S3). Sanger sequencing detects 16 mutations that correspond to sites with prior low coverage (< 5 reads). This suggests a false negative rate of 16/(172 + 16) = 8.51%, approximately in accord with the above simulation in near/non-duplicates but lower than that within duplicates.

We take the FNR rate into account when estimating mutation rates. We first normalized observed numbers as “number of mutations identified”/(1-“regional FNR”), FNR being specific to each ascus, FNR expressed as a fraction. The per genome mutation rate for each cross was the calculated as “average number of normalized mutations per ascus”/“2 source parents,” and the per bp rate was calculated as “normalized per genome rate”/“reference genome size”. On average, there are 135.3 ± 21.4 (sem) base substitution mutations detected with a 2:2 segregating ratio, equivalent to 3.34 × 10^−6^ ± 5.29 × 10^−7^ (sem) per site per sexual generation (Table 1).

While we map by reference to the published genome, we also examine genomic domains with twice the expected read coverage under the supposition that these are recent spontaneous duplications. Putative spontaneous duplicates during sexual cycle were identified first by searching for 2× duplicates only present in each ascus but not present in parental strains and with a ratio of 2:2. Another approach, DELLY [40], which integrates paired-end and split-read analysis, was then applied to confirm the initial candidates, and only those could be confirmed by DELLY were retained in the final results. These appear to be rare (no more than 1% of sequence, Additional file 2: Datasheet S4).

### Definition of duplicate sequences

Our default method to define duplicate sequences uses a criterion of over 65% identity [19] and at least 100 bp of alignable sequence [6] (Additional file 1: Figure S2, Table S3, and Additional file 2: Datasheet S4). This method for defining what constitutes a duplicated region in the eyes of RIP appears to be efficient, meaning a high fraction of the percentage of 2:2 mutations called for the percentage of genomic sequence identified (Additional file 1: Table S3).

This was chosen as the default as it performed the best. In total, we investigated four possible methods of definition. As above duplicates can be classified depending on Blast search (the 65% identity and 100 bp alignable length used above), which we term the Dup-Blast method, or as regions with 2× sequencing depth on average (Dup-Depth), or as regions carrying “heterozygous” alleles (a signature of mis-mapping, Dup-Het), or as regions that follow the matching period defined by Kleckner [6, 20] (Dup-Period, a matching period of 10 ~ 12 were used here) (Additional file 1: Table S3). All other methods in isolation identify a lower percentage of mutations while describing a higher proportion of genomic sequence (Additional file 1: Figure S2 and Table S3). Amalgamation of all these signatures would suggest at most 40% of the genome (16 Mb) could be “duplicated,” while the remaining 60% is not. Strikingly, the proportion of 2:2 mutations goes from 87.4% for Dup-Blast to just 92.3% in this 40%, suggesting the 16% of the genome called as Dup-Blast captures efficiently the proportion of the genome that RIP considers to be duplicated. More specifically, we found about 32.7% of 2:2 mutations previously defined as non-duplicate associated mutations (i.e., non-Dup-Blast) could be assigned to Dup-Depth/Het/Period domains (Additional file 2: Datasheet S2).

### Permutation test for mutation clusters

A mutation cluster was defined as having at least two mutations within 1 kb in a single haploid genome (Additional file 1: Figure S4). As each mutation cluster, especially those within duplicates, usually only consists of exclusively C→T or exclusively G→A mutations, they were most likely raised from a single round of RIP. To test whether the number of “clustered mutations” in duplicates were different from random distributions, we re-distributed the mutations at random within the 16% of the genome that is duplicate, picking randomly selected G:C sites within these domains in each haploid genome from the same ascus. For each simulation, we counted the number of mutations that accord with the definition of a cluster. The *P* value (expected type I error rate) was derived from 10,000 randomizations as (*n* + 1)/(*m* + 1), where *n* is the number of observations with as many or more clustered mutations than that observed and *m* is the number of randomization. We employed this method to ask whether there are more clusters within the duplicate domains given the positions of G and C residues within the duplicate domains.

As we define clusters based exclusively on the locations of mutations observed in the very same haploid genome, it is possible that clusters identified in one spore may overlap with clusters identified in a different spore. The extent of overlap of clusters (Additional file 1: Figure S4) was tested by shuffling all clusters within duplicates among tetrads using BEDTools [41] “shuffle” command. Ten thousand randomizations were performed to obtain the *P* value, as (*n* + 1)/(*m* + 1), where *n* is the number of observations with the same number or more mutations seen in randomized overlapping clusters than that observed in the real clusters and *m* is the number of randomizations. The related pipelines are available at https://github.com/wl13/Neurospora_mutation. All statistical tests were performed in R [42].

### Estimation of *N*_e_

For the estimation of effective population size, we employed the compendium of heterozygosity (*π*) measures provided by Lynch et al. [1]. We employed their compendium of mutation rates (*μ*) in addition to ours. *N*_e_ was then defined as *N*_e_ = *π/ μ D* (*1 – π*), where *D* is the ploidy adjustment (2 for haploid species, 4 for diploids). The mutation rates in CDS were estimated directly by us for *Neurospora* (rather than extrapolated from genomic mutation rates scaled to the proportion of sequence that is CDS) but otherwise taken from this prior compendium. The revised data table is presented as Additional file 1: Table S7.

### Analysis of features of duplicate regions

Information about the centromeric regions was obtained from Smith et al. [43]. Lengths, GC contents and best BLAST identities for different duplicate regions (Dup-Blast) are summarized in Additional file 1: Table S8. To investigate whether a certain region has undergone RIP, the “RIP index” method [44] was applied with a threshold of TpA/ApT > 2 or (CpA + TpG)/(ApC + GpT) < 0.7 [19]. Hi-C data from Galazka et al. [45] was used to search for interacting regions after dissecting the genomes into 10 kb windows (Additional file 1: Table S9).

## Supplementary information

**Additional file 1: Supplementary Notes. Figure S1.** Spectra of 2:2 mutations within, near or outside of duplicates. **Figure S2.** Defining duplicates by different approaches. **Figure S3.** Distance between nearest two 2:2 mutations within duplicates. **Figure S4.** Clustered mutations with a strand-biased. **Figure S5**. Tri-nucleotide content in duplicates. **Figure S6**. Sanger verification of identified mutations. **Table S1.** Spectra of mutations in *N. crassa*. **Table S2.** Parental source of identified 2:2 mitotic mutations. **Table S3.** Genomic coverage and enclosed mutations in duplicates defined by different approaches to define “duplicate”. **Table S4.** Overview of identified mutation clusters within duplicates among all sexual crosses. **Table S5.** Approximate genomic positions of putative centromeric regions. **Table S6.** Number of mutations per genome employing the least generous (most conservative) definition of non-duplicates. **Table S7.** Cross species estimates of mutation rates and related parameters. **Table S8.** Summary features of Dup- Cl, One, and Zero regions. **Table S9.** Windows with putative Hi-C interactions.

**Additional file 2: Datasheet S1.** Mapped depth and genome coverage of sequenced samples. **Datasheet S2.** Identified mutations during sexual and asexual cycles of ***N. crassa***. **Datasheet S3.** Sanger verification of randomly picked mutations. **Datasheet S4.** Duplicates in ***N. crassa*** identified through different approaches. **Datasheet S5**. Clusters formed by 2:2 mutations in sexual cycle of *N. crassa*. **Datasheet S6.** False negative rates estimated using simulation approach. **Datasheet S7.** Detailed properties of Dup-Cl, Dup-One, and Dup-Zero regions. **Datasheet S8.** Tri-nucleotide context within different duplicates of ***N. crassa*** genome.

**Additional file 3.** Review history.

## Data Availability

The sequencing reads are deposited at the National Center for Biotechnology Information (NCBI) under BioProject PRJNA373800 [46] and PRJNA553108 [47]. All related codes are available at GitHub page https://github.com/wl13/Neurospora_mutation as well as 10.5281/zenodo.3837458 [48].

## Abbreviations

RIP: Repeat-induced point mutation
CDS: Coding sequence
TE: Transposable element
MSUD: Meiotic silencing by unpaired DNA
MIP: Methylation induced premeiotically
SIS: Sex-induced silencing
