# Supplementary material for: Repeat-induced point mutation in Neurospora crassa causes the highest known mutation rate and mutational burden of any cellular life
Source: Genome Biol. 2020 Jun 16;21:142. doi: 10.1186/s13059-020-02060-w (PMC7296669; doi:10.1186/s13059-020-02060-w)
Supplement: Supplementary file 1 — Additional file 1: Supplementary Notes. Figure S1. Spectra of 2:2 mutations within, near or outside of duplicates. Figure S2. Defining duplicates by different approaches. Figure S3. Distance between nearest two 2:2 mutations within duplicates. Figure S4. Clustered mutations with a strand-biased. Figure S5. Tri-nucleotide content in duplicates. Figure S6. Sanger verification of identified mutations. Table S1. Spectra of mutations in N. crassa. Table S2. Parental source of identified 2:2 mitotic mutations. Table S3. Genomic coverage and enclosed mutations in duplicates defined by different approaches to define “duplicate”. Table S4. Overview of identified mutation clusters within duplicates among all sexual crosses. Table S5. Approximate genomic positions of putative centromeric regions. Table S6. Number of mutations per genome employing the least generous (most conservative) definition of non-duplicates. Table S7. Cross species estimates of mutation rates and related parameters. Table S8. Summary features of Dup- Cl, One, and Zero regions. Table S9. Windows with putative Hi-C interactions. [file 13059_2020_2060_MOESM1_ESM.docx]

# Additional file 1

**Supplementary Notes**

Comparison of mutation rate estimated in this study with prior studies ………… 2

Mutations with a 3:1 ratio in sexual cycle of *N. crassa* …………………………… 3

Different duplicates have different RIP rates …………………………………. 3

**List of Supplementary Figures**

Figure S1. Spectra of 2:2 mutations within, near or outside of duplicates in the sexual cycle. ……………………………………………………………………………. 7

Figure S2. Defining duplicates by different approaches. …………………………. 8

Figure S3. Distance between the nearest two 2:2 mutations within duplicates. .…. 9

Figure S4. Clustered mutations with a strand-bias. …………………………..…. 10

Figure S5. Tri-nucleotide content in duplicates. ……………………………….…. 11

Figure S6. Sanger verification of identified mutations. …………………….…. 12

**List of Supplementary Tables**

Table S1. Spectra of mutations in N. crassa. …………………………………. 14

Table S2. Parental source of identified 2:2 mitotic mutations. ……………………. 15

Table S3. Genomic coverage and enclosed mutations in duplicates defined by different approaches to define “duplicate’. ……………………………..…………………. 16

Table S4. Overview of identified mutation clusters within duplicates among all sexual crosses. ……………………………………………………………….………. 17

Table S5. Approximate genomic positions of putative centromeric regions. ….…. 18

Table S6. Number of mutations per genome employing the least generous (most conservative) definition of non-duplicates. ……….……………………………. 19

Table S7. Cross species estimates of mutation rates and related parameters. ……... 20

Table S8. Summary features of Dup- Cl, One, and Zero regions …………..……. 21

Table S9. Windows with putative Hi-C interactions. ………………………………. 22

**Supplementary References** ………………………………….…………………. **23**

# Supplementary Notes

## Comparison of mutation rate estimated in this study with prior studies

The prior estimation of mutation rate for *N. crassa* by Lynch and colleagues [1,2] was assembled from a range of locus-based studies. A brief summary of each study they used is given below.

1) Single-site reversion (to histidine utilization) rate estimated by incubating each strain colony to 10^7^ conidia suspension and count the His^+^ colonies [3]. The estimated rate is 10.5 × 10^-9^ revertant per conidium.

2) Rate estimated by measuring forward mutations at the *mtr* locus encoding neutral amino acid permease (scoring only uninucleate conidia) [4]. The estimated rate is 3.0 × 10^-7^ revertant per nucleus, and was converted by Lynch [1] to a per site rate of 3.0 × 10^-7^ × 0.324 (32.4% of assayed mutations being base substitutions) / (1413 bp × 0.143 detectability) = 0.48 × 10^-9^.

3) Mutations detected at *mtr* gene during the sexual cycle [5]. This study detects a total of 42 spontaneous mutations by *mtr* gene, from which Lynch estimated a frequency of 7.0 × 10^-7^, which he converts to a rate of [7.0 × 10^-7^ × 0.324 / (1413 × 0.143) =] 1.12 × 10^-9^ per site per sexual cycle. However, since this locus is not a target designed for RIP [5], this rate is likely to reflect a non-RIP rate. Another locus described by Watters and Stadler was in a mutant pA24. Here they found a total of 48 C->T transitions (all C->T were on the sense strand) in 1-kb region, which might be subject to only a single round of RIP [5]. This RIPed data was not used in Lynch’s estimation.

4) Estimation of the mutant frequencies (number of mutants/viable conidium) for both *mtr* (2 × 10^-7^) and *trp-2* (1.1 × 10^-9^) loci [6]. From this Lynch estimated a per site rate of [2.0 × 10^-7^ × 0.324 / (1413 × 0.143)] = 0.32 × 10^-9^, and 1.1 × 10^-9^ × 3 = 3.3 × 10^-9^ for *mtr* and *trp-2*, respectively.

5) From Sakai et al.’s mutation data at the *ad-3A* locus [7], Lynch estimated a rate of 2.4 × 10^-7^ adenine prototrophs/survivors, and converted it to a per site rate of 2.4 × 10^-7^ × 0.82 (82% of mutations appear to be point changes) × 3.55 (correct for limited abilities to detect mutations in sectoring colonies) / (924 bp × 0.143) = 5.28 × 10^-9^.

6) From de Serres et al.’s mutation data at the *ad-3* region [8], Lynch estimated a rate of 1.39 × 10^-7^ adenine prototrophs/survivors, and converted to a per site rate of 1.39 × 10^-7^ × 0.82 × 3.55 / (924 × 0.143) = 3.06 × 10^-9^.

Note that except the third estimation, all other estimates were from asexually propagated lines. Even in the third estimation, the rate seems to include no RIP mutations. Therefore, the unit of Lynch and colleagues’ estimation is likely to be per site per few days (usually 3~7 days to incubate the conidia in one situation and then changed to another situation, and count the mutants afterwards) [1,2]. In this sense, assume a short length of 3 days, the 4.66 × 10^-9^ per site per 3-day rate without RIP (or 1.55 × 10^-9^ per site per day rate) is comparable to the 9.05 × 10^-9^ per site per day estimated for the asexual lines in this study or is more similar to the 6.79 × 10^-9^ per coding site per day rate during asexual propagation.

## Mutations with a 3:1 ratio in sexual cycle of *N. crassa*

The correction based on 3:1 mutations is a conservative correction as new 2:2 mutations can also undergo meiotic gene conversion and because many 3:1 events likely reflect late resolved RIP. Indeed, these tend to in duplicated sequence and tend to predominantly be C->T/G->A transitions, typical of RIP (Additional file 1: Table S1). Similar to 2:2 mutations, higher mutation rates within or near duplicates were found for 3:1 mutations (Table 1). There was also a high proportion of C->T/G->A mutations in 3:1 mutations (Additional file 1: Table S1), especially related to duplicates. Around 52.8% of 3:1 mutations were found to be clustered C->T/G->A changes (78.9% within duplicates defined by Dup-Blast, 47.1% in near duplicates, and 47.4% in non-duplicates). In RIP knockout lines we found no 3:1 mutations. To be conservative, however, we presume that 3:1 mutations are not RIP associated.

## Different duplicates have different RIP rates

As mentioned in main text, the most majority of mutations (>70%) in duplicates were clustered within approximately 800kb ranges, far more than which could be expected from random distributions (permutation test with 10,000 replicates, expected mutations in clusters = 241, observed = 6,072, *P* = 0.0001). The remaining over 5Mb duplicated parts contain only ~2,000 mutations (~30%), suggesting that neither all duplicates nor the full range of certain duplicated regions are equally affected by RIP. Of those mutation clusters, over a third of ranges (~310kb in size) were found to overlap in at least two tetrads among all crosses, which significantly enriched ~80.0% of clustered mutations (Chi-squared with Yates correction = 3801.6, *P* = 0). Reposition of those mutation clusters within duplicates through randomization suggested the extent of overlap could not be explained under a null model (permutation test with 10,000 replicates, expected mutations in overlapped clusters = 2,476, observed = 4,911, *P* = 0.0001).

The observation of clustered mutations within only small part of duplicates (Additional file 2: Datasheet S5) would suggest varied RIP efficiency even within duplicates. The 800kb mutation clusters resided within 241 duplicates (duplicates with clustered mutations, hereafter denoted as “Dup-Cl”), spanning a total of 2.16 Mb (Additional file 1: Table S8 and Additional file 2: Datasheet S7). This size was similar to the total size of duplicates when we consider only those with singleton mutations (denoted as “Dup-One”), and was also similar to the overall size of those duplicates with no mutations (denoted as “Dup-Zero”). Dup-Cl were generally larger in size (contained within 241 duplicated blocks with a median length of 6,400bp), in contrast, Dup-Zero were found to be rather fragmented (contained within 3,826 blocks with a median length of 200bp), suggesting that the length of duplicates might be an important factor to trigger RIP.

The canonical threshold suggested only duplicates with ≥ 80% identity and ≥ 400bp alignable DNA length could be RIPed [9,10]. We found most regions in Dup-Cl and Dup-One match well with this threshold, and over 1 Mb of Dup-Zero could also pass this threshold (Additional file 1: Table S8 and Additional file 2: Datasheet S7). To get rid of the concern that some regions lack RIP merely as they do not meet the basic requirements, we applied the more stringent criteria to filter all three types of duplicates and only use those post-filtered ones in subsequent analyses.

Despite the fact the RIP mutates cytosines, the duplicates that were subject to RIP have a significantly lower GC content (overall GC content = 31.1%) than the Non-Dup (GC content = 51.6%, Chi-squared with Yates correction = 4.92 × 10^5^, *P* = 0). Similar to a difference of GC content between duplicates and non-duplicates, the GC content was found to increase in the order: Dup-Cl (28.0% ± 2.8%), Dup-One (28.9% ± 3.8%) and Dup-Zero (32.3% ± 9.0%) (Additional file 1: Table S8 and Additional file 2: Datasheet S7), suggesting that the lower the local GC content, the higher the RIP efficiency, despite there being fewer C residues to mutate.

This observation has several possible explanations, e.g., 1) RIP favors Dup-Cl regions because they have special nucleotide context or other properties possibly independent of the GC content itself; 2) all Dup-Cl, One, and Zero regions were originally equally affected by RIP but Dup-One and Dup-Zero became resistant to RIP due to some factors such as reduced sequence similarity or fewer duplicated copies or etc. We used the well-established “RIP index” method [11] to investigate whether the three regions have already undergone RIP.

Using a threshold of TpA/ApT > 2 or (CpA + TpG)/(ApC + GpT) < 0.7 [10], the RIP indices predicted 92.4%, 90.9% and 83.3% portions of Dup-Cl, One, and Zero regions, respectively (Additional file 2: Datasheet S7), as having been RIP-mutated. These portions are remarkably higher than that predicted for Non-Dups, which is only 2.2%, consistent with the suggestion that most of the duplicates in *N. crassa* have been mutated RIP [10]. The Dup-Cl and One have a significantly higher proportion of RIP-mutated regions than Dup-Zero (Chi-squared test with Yates' correction, *P* < 1.7 × 10^-6^), while no significant differences were found between Dup-Cl and One (Chi-squared test with Yates' correction, Chi-square = 1.4, *P* = 0.242). These results thus defied the assumption that only Dup-Cl regions were preferentially targeted by RIP in the past, and suggest instead that all three types of duplicates are RIP relics but behave differently as regards new RIP events.

Analyses of tri-nucleotide components suggested all three regions have similar enrichment of AT-only triplets, like ATA, TAT, etc., which account for 42.9%, 42.8%, 41.0% in Dup-Cl, One, and Zero regions, respectively (Additional file 1: Figure S5 and Additional file 2: Datasheet S8). No such enrichment was found in non-duplicates, which only have 9.1% of AT-only triplets. The highly similar pattern of tri-nucleotide context in three regions (Spearman’s rank correlation rho > 0.99, *P* < 2.2 × 10^-16^) would suggest they are not so different in their nucleotide contexts.

To find out why the three regions differed in current RIP efficiency, we further analyzed the properties of different copies of a duplicate (Additional file 1: Table S8 and Additional file 2: Datasheet S7). In terms of best matched identity in each region, we found higher but only subtle different identities in Dup-Cl regions (85.51% ± 4.19%) compared to Dup-One (84.39% ± 4.83%) and Dup-Zero (85.30% ± 5.56%). A similar trend was found in terms of copy numbers in each region with the highest in Dup-Cl, and lowest in Dup-Zero (Additional file 1: Table S8 and Additional file 2: Datasheet S7), but still only with marginal differences. The not so different properties of three types of duplicates would suggest a putative but rather weak influence of the identity and copy numbers to RIP.

As RIP requires the pairing of duplicated sequences, the higher RIP rate found in Dup-Cl would suggest these regions could have higher chances interacting with each other. We confirmed this using the Hi-C data [12] by searching for interacting regions after dissecting the genomes into 10kb windows (Additional file 1: Table S9). It was found that around 44.6% windows in Dup-Cl could have interactions with other Dup-Cl windows (Expected = 14.6% assume equal chance of one window to be interacted with another one in the genome, Chi-squared with Yate’s correction = 253.0, *P* < 2.2e-16), which is apparently higher than 32.4% between Dup-One and Dup-One (Expected = 13.6%, Chi-squared with Yate’s correction = 97.1, *P* < 2.2e-16) and 31.6% between Non-Dup and Non-Dup (Expected = 62.5%, Chi-squared with Yate’s correction = 614.3, *P* < 2.2e-16). The Dup-Zero have the fewest interactions between each two windows from these regions, which is not significant different from the null expectation (Observed = 7.6%, Expected = 9.3%, Chi-squared with Yate’s correction = 0.062, *P* = 0.804).

Besides the interactions between windows from the same type of duplicates, i.e., Dup-Cl vs Dup-Cl, Dup-One vs Dup-One, and Dup-Zero vs Dup-Zero, 42.7% of Dup-One windows were found to interact with windows from Dup-Cl (Expected = 14.6%, Chi-squared with Yate’s correction = 208.3, *P* < 2.2e-16), while in contrast, only 21.8% of Dup-Zero windows (Expected = 14.6%, Chi-squared with Yate’s correction = 8.86, *P* = 0.00292) and 9.7% of Non-Dup windows could interact with Dup-Cl (Expected = 14.6%, Chi-squared with Yate’s correction = 28.0, *P* = 1.19e-7). Therefore, it’s possible that Dup-Cl are more susceptible to RIP owing to their ability to frequently interact with most duplicates especially those also from Dup-Cl (Expected = 38.9% within Dup-Cl among all duplicates, Chi-squared with Yate’s correction = 40.0, *P* = 2.6e-10). For Dup-One, the interactions between Dup-One and Dup-Cl seem to be higher than between Dup-One to other duplicates (Expected = 38.9% between Dup-One and Dup-Cl, Chi-squared with Yate’s correction = 20.3, *P* = 6.74e-6), suggesting their mutations might also be a result by RIP but they are more likely to trigger RIP in Dup-Cl when interact with Dup-Cl regions. These trends are unchanged even if we exclude centromere regions, which were supposed to have the strongest interactions [12] and mainly consists of Dup-Cl and Dup-One.

# Supplementary Figures


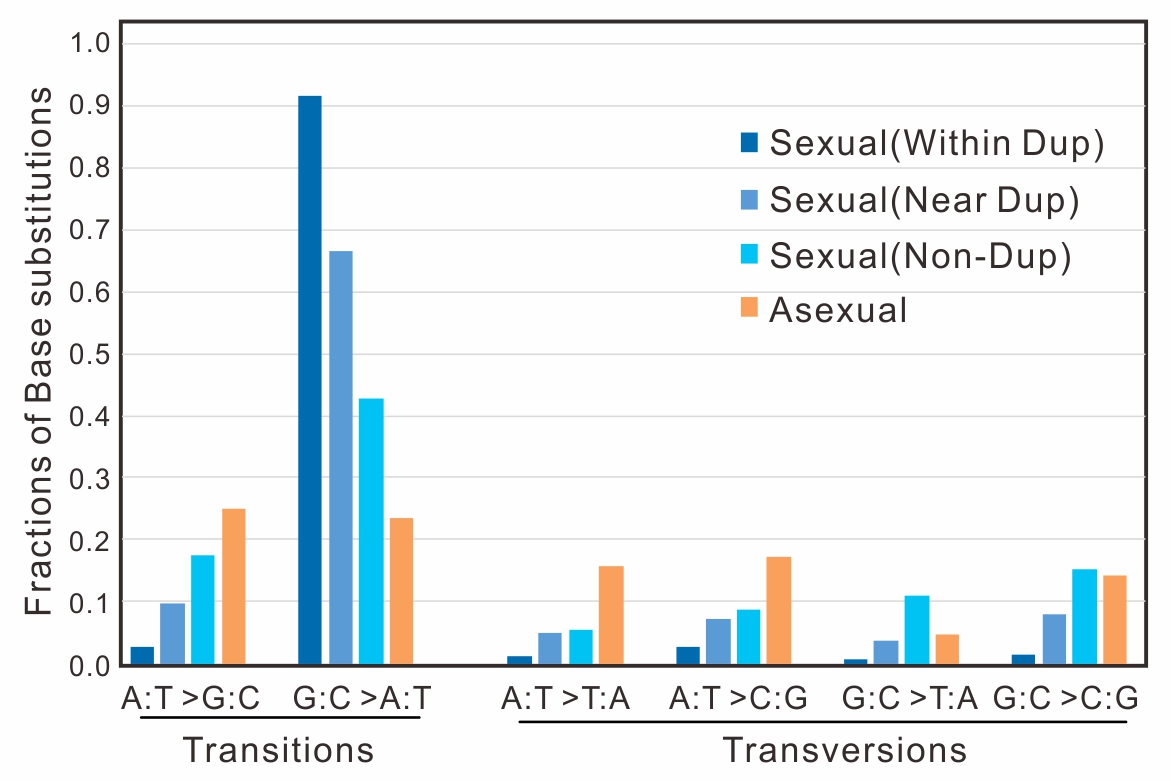


## Figure S1. Spectra of 2:2 mutations within, near or outside of duplicates in the sexual cycle. Additionally the spectrum of mutations from the asexual cycle are compared here.


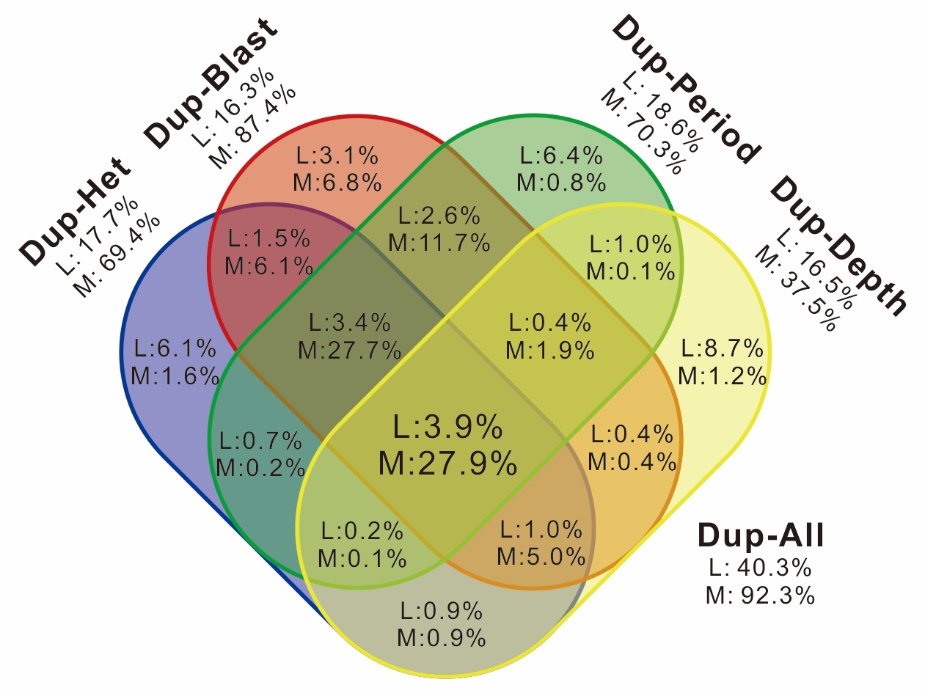


## Figure S2. Defining duplicates by different approaches. Both genomic coverage (L = duplicate length / genomic length) and enclosed mutations (M = enclosed 2:2 mutation sites / all 2:2 mutation sites) are presented as proportions. The overall genomic coverage and enclosed 2:2 mutation sites are given below each approach, e.g., Dup-Blast, Dup-Period. A table summarizing all these numbers can be found in Additional file 1: Table S3.


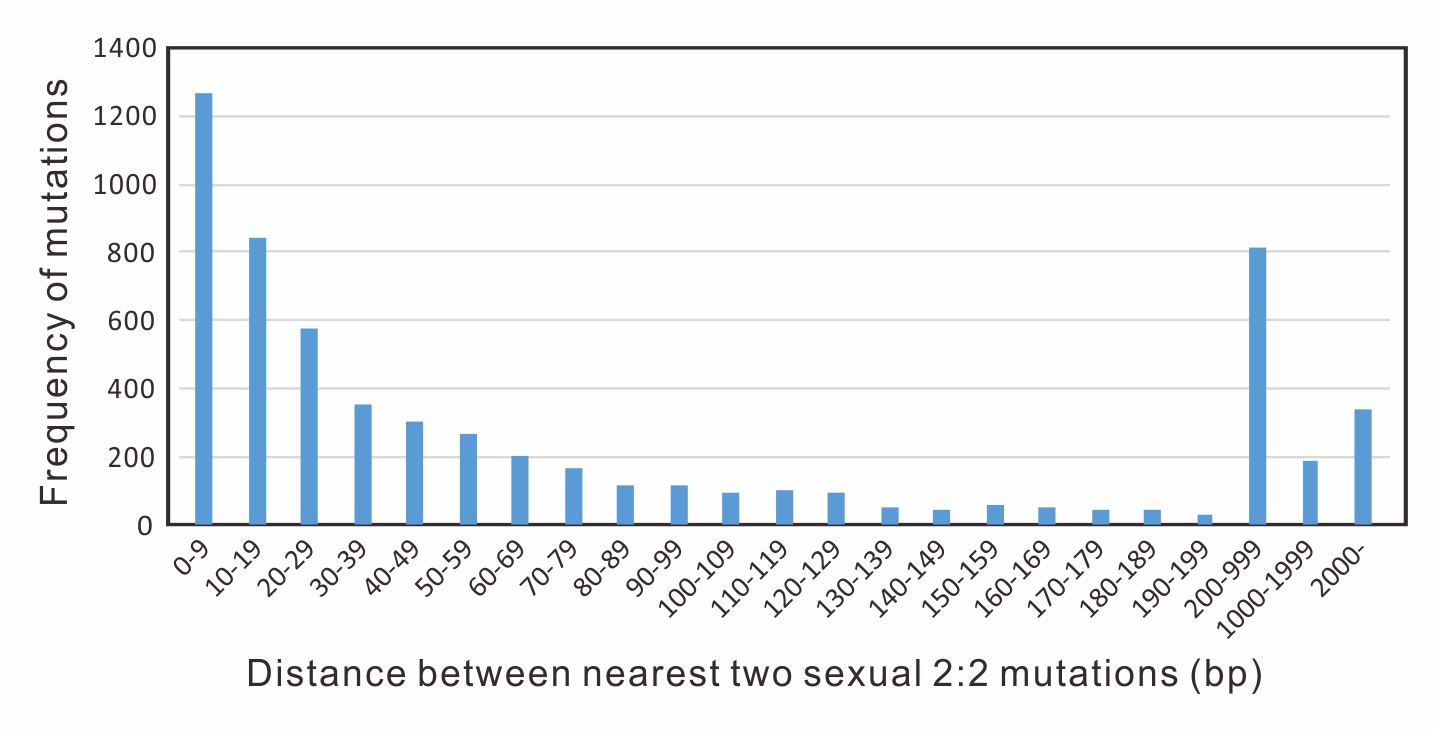


## Figure S3. Distance between the nearest two 2:2 mutations within duplicates.


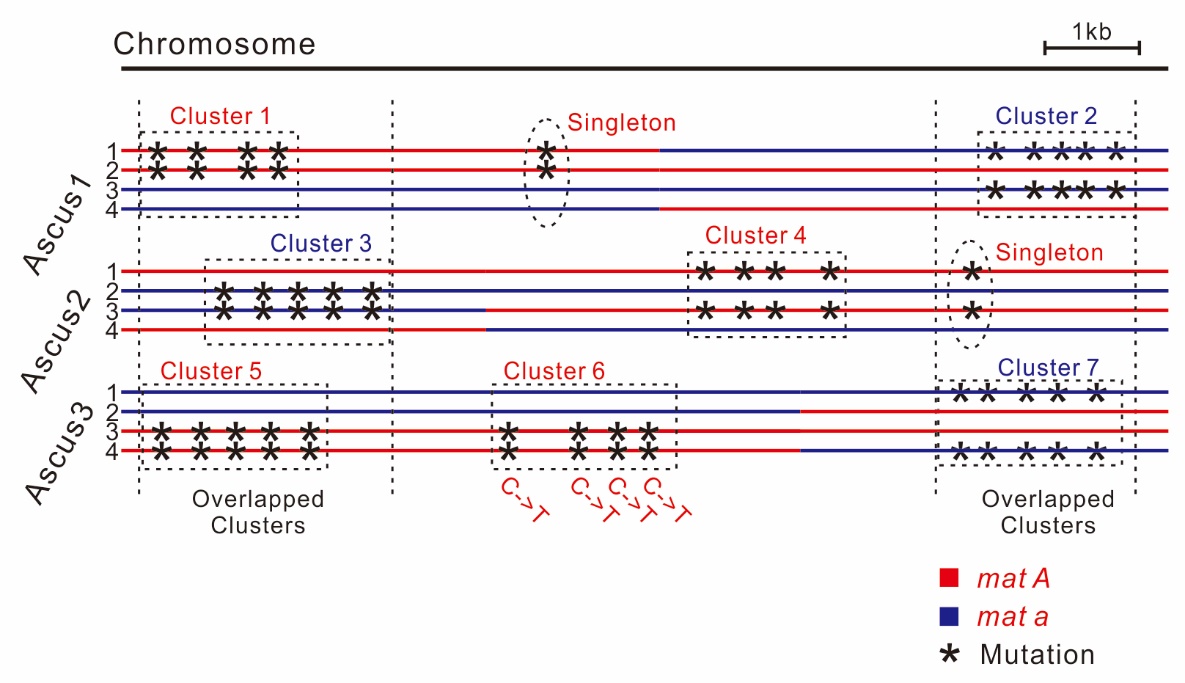


## Figure S4. Clustered mutations with a strand-bias. The clustered mutations in duplicates (defined as Dup-Cl) were almost exclusively C->T (or exclusively G->A) mutations. A certain number of clusters were found to overlap between different asci within or between crosses.


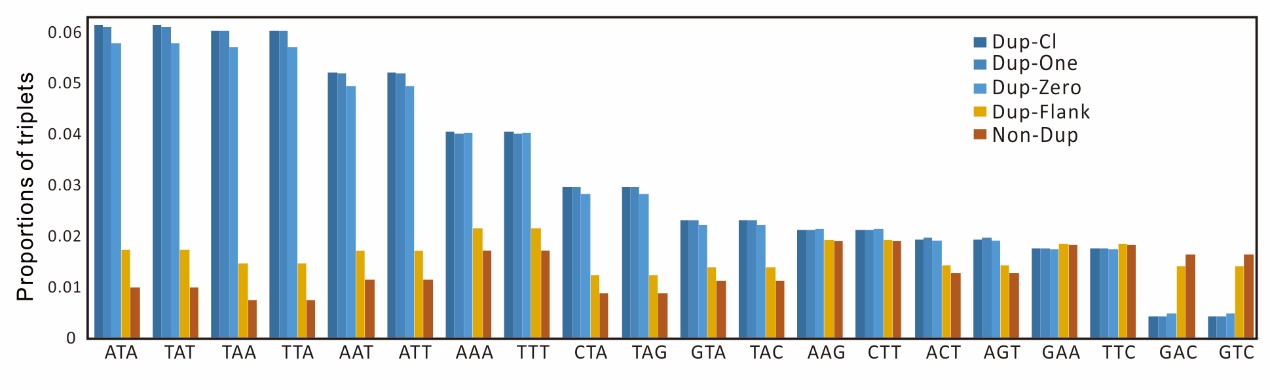


## Figure S5. Tri-nucleotide content in duplicates. The duplicates (Dup-Blast) could be further divided into Dup-Cl (duplicates with clustered mutations), Dup-One (duplicates with only singleton mutations), and Dup-Zero (duplicates without mutations). All duplicates have a similar pattern of triplet contexts which are different from the non-duplicates (non-Dup). No enrichment of GAC or GTC triplets was found in duplicates. Only the 18 triplets with the highest proportion in Dup-Cl, as well as GAC and GTC triplets, are shown here.


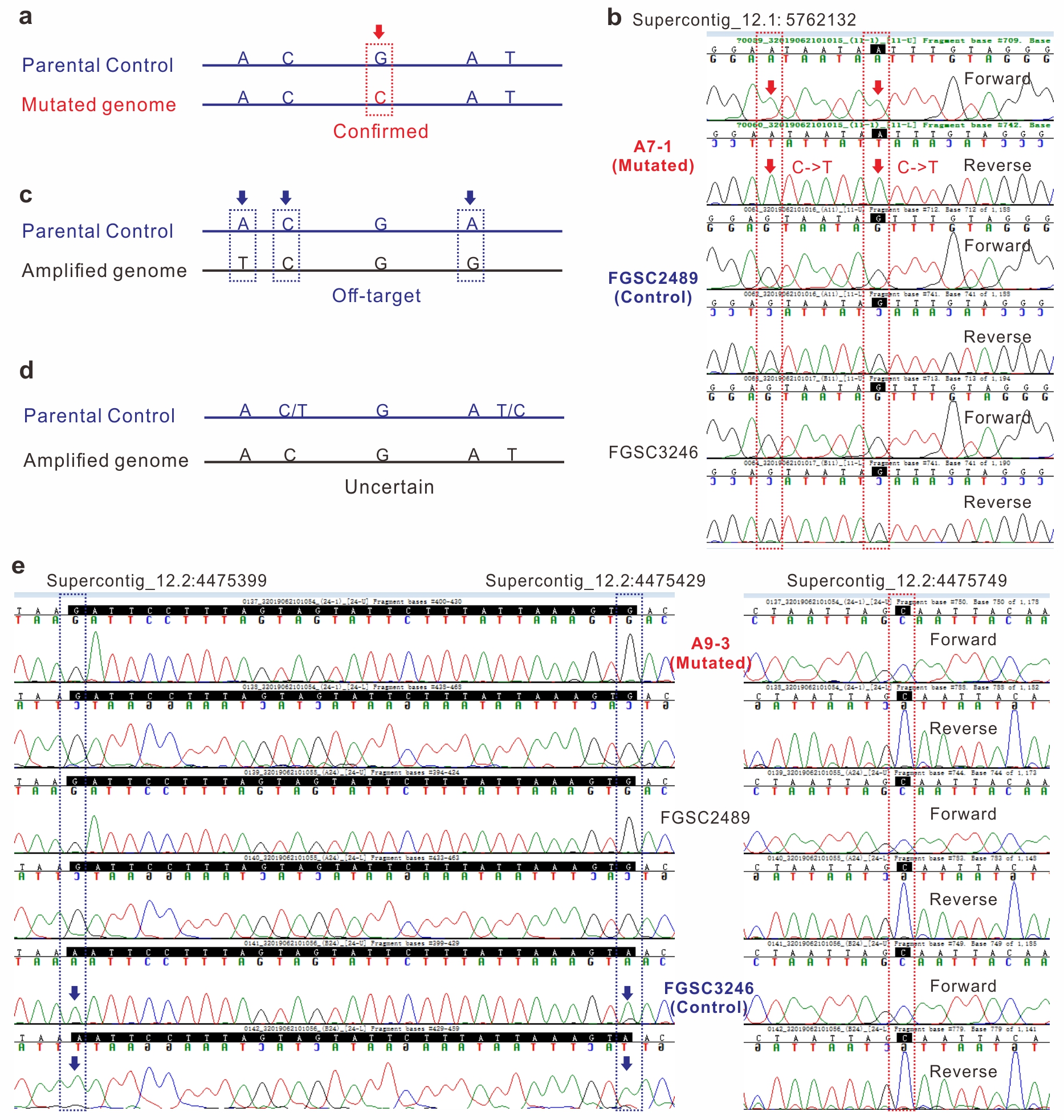


## Figure S6. Sanger verification of identified mutations. (a) A mutation would be confirmed if the mutated allele was present only in the mutated sample, i.e. not in parental control. (b) An example of two verified mutations from ascospore A7-1. Two C->T mutation sites (at genomic location Supercontig_12.1:5762132 and 5762138) were confirmed here. (c) Off-target case where only non-mutated DNA fragment was amplified. This could be determined through inspection of nearby polymorphic sites. (d) Uncertain case where no polymorphic sites could be found nearby. These cases were usually accompanied by “heterozygous” chromatograms in the parental control, which also indicates amplification of non-target DNA fragments. (e) An example of the Off-target case. A mutation was supposed to be present in A9-3 (right panel, genomic location Supercontig_12.2:4475749, C->T mutation), but not found in Sanger sequencing (red rectangular). However, as this mutation was supposed to be raised from the FGSC3246 background (distinguishable by two A/T sites nearby, marked by blue rectangular and arrows), the mutated haplotype was actually not amplified/sequenced. The mutation sites were marked by dashed rectangular and red arrows. The polymorphism sites were marked by blue rectangular and blue arrows.

# Supplementary Tables

## Table S1. Spectra of mutations in *N. crassa*.

| Type of mutations | 2:2 mutations | | | 3:1 mutations | | | Asexual mutations | | |
| --- | --- | --- | --- | --- | --- | --- | --- | --- | --- |
|  | **Dup** | **Near Dup** | **Non-Dup** | **Dup** | **Near Dup** | **Non-Dup** | **Dup** | **Near Dup** | **Non-Dup** |
| Transitions (Total) | 6,394 (0.943) | 172 (0.764) | 454 (0.601) | 68 (0.971) | 18 (0.947) | 16 (0.842) | 13 (0.394) | 2 (0.333) | 16 (0.64) |
| T->C/A->G | 173 (0.026) | 22 (0.098) | 131 (0.173) | 1 (0.014) | 0 (0) | 0 (0) | 8 (0.242) | 1 (0.167) | 7 (0.28) |
| C->T/G->A | **6,221 (0.918)** | **150 (0.667)** | **323 (0.427)** | **67 (0.957)** | **18 (0.947)** | **16 (0.842)** | **5 (0.152)** | **1 (0.167)** | **9 (0.36)** |
| Transversions (Total) | 385 (0.057) | 53 (0.236) | 302 (0.399) | 2 (0.029) | 1 (0.053) | 3 (0.158) | 20 (0.606) | 4 (0.667) | 9 (0.36) |
| A->T/T->A | 71 (0.01) | 11 (0.049) | 41 (0.054) | 1 (0.014) | 1 (0.053) | 0 (0) | 6 (0.182) | 0 (0) | 4 (0.16) |
| A->C/T->G | 172 (0.025) | 16 (0.071) | 65 (0.086) | 1 (0.014) | 0 (0) | 1 (0.053) | 6 (0.182) | 3 (0.5) | 2 (0.08) |
| C->A/G->T | 47 (0.007) | 8 (0.036) | 82 (0.108) | 0 (0) | 0 (0) | 1 (0.053) | 2 (0.061) | 0 (0) | 1 (0.04) |
| C->G/G->C | 95 (0.014) | 18 (0.08) | 114 (0.151) | 0 (0) | 0 (0) | 1 (0.053) | 6 (0.182) | 1 (0.167) | 2 (0.08) |
| A/T sites | 416 (0.061) | 49 (0.218) | 237 (0.313) | 3 (0.043) | 1 (0.053) | 1 (0.053) | 20 (0.606) | 4 (0.667) | 13 (0.52) |
| G/C sites | 6,363 (0.939) | 176 (0.782) | 519 (0.687) | 67 (0.957) | 18 (0.947) | 18 (0.947) | 13 (0.394) | 2 (0.333) | 12 (0.48) |
| Total | 6779 (1) | 225 (1) | 756 (1) | 70 (1) | 19 (1) | 19 (1) | 33 (1) | 6 (1) | 25 (1) |

The numbers outside the parenthesis represent the identified mutation sites, while the numbers within parenthesis represent fractions of each type of mutations. For sexual cycle, both 2:2 and 3:1 mutations displayed extreme skewed spectra towards C**-**>T/G->A (in bold), especially within duplicates. No such strong skewness observed in asexual lines. Dup: within duplicates (defined by Dup-Blast), Near Dup: near duplicates (400bp upstream and downstream flanking regions), Non-Dup: non-duplicate

## Table S2. Parental source of identified 2:2 mitotic mutations.

| **Cross** | **Parent (Mating type)** | **Average genomic proportions in sequenced asci** | **No. of 2:2 mutations** | | | |
| --- | --- | --- | --- | --- | --- | --- |
|  |  |  | **Within Dup** | **Near Dup** | **Non-Dup** | **Total** |
| A | P1 (*mat A*) | 37.2% | 409 | 12 | 27 | 448 |
|  | FGSC3246 (*mat a*) | 37.0% | 474* | 34** | 88*** | 596*** |
| B | FGSC1363 (*mat A*) | 49.3% | 337 | 31 | 86 | 454 |
|  | FGSC4200 (*mat a*) | 48.9% | 667*** | 49 | 280*** | 996*** |
| C | FGSC2225 (*mat A*) | 49.8% | 9 | 8 | 53 | 70 |
|  | FGSC3246 (*mat a*) | 49.4% | 652*** | 51*** | 454*** | 1157*** |
| E | FGSC2489 (*mat A*) | 34.1% | 1008 | 47 | 68 | 1123 |
|  | FGSC4200 (*mat a*) | 34.9% | 1385*** | 39 | 100* | 1524*** |
| G | C14-2 (*mat A*) | 37.5% | 661 | 33 | 122 | 816 |
|  | C14-4 (*mat a*) | 37.2% | 1075*** | 36 | 257*** | 1368*** |

Mutations from parental strain with *A* mating-type (*mat A*) are significantly more than from parental strain with *a* mating-type (*mat a*), Chi-squared test with Yates' correction, * *P* < 0.05, ** *P* < 0.01, *** *P* < 0.001. However whether this reflect mating type or strain differences is unresolved

## Table S3. Genomic coverage and enclosed mutations in duplicates defined by different approaches to define “duplicate’.

| Methods | Length (bp) | Length (%) | No. of 2:2 mutation sites | % of 2:2 mutation sites | No. of 1:3 mutation sites | % of 1:3 mutation sites | Enrichment |
| --- | --- | --- | --- | --- | --- | --- | --- |
| Blast, Depth, Het, Period | 1,571,622 | 3.9% | 2167 | 27.9% | 8 | 7.4% | 7.19 |
| Blast, Depth, Het | 412,078 | 1.0% | 387 | 5.0% | 11 | 10.2% | 4.90 |
| Blast, Depth, Period | 155,179 | 0.4% | 145 | 1.9% | 0 | 0.0% | 4.87 |
| Blast, Het, Period | 1,365,885 | 3.4% | 2146 | 27.7% | 15 | 13.9% | 8.19 |
| Depth, Het, Period | 61,529 | 0.2% | 5 | 0.1% | 0 | 0.0% | 0.42 |
| Blast, Depth | 147,921 | 0.4% | 29 | 0.4% | 1 | 0.9% | 1.02 |
| Blast, Het | 601,060 | 1.5% | 471 | 6.1% | 10 | 9.3% | 4.09 |
| Blast, Period | 1,072,117 | 2.6% | 909 | 11.7% | 7 | 6.5% | 4.42 |
| Depth, Het | 382,671 | 0.9% | 72 | 0.9% | 2 | 1.9% | 0.98 |
| Depth, Period | 397,346 | 1.0% | 9 | 0.1% | 1 | 0.9% | 0.12 |
| Het, Period | 300,461 | 0.7% | 14 | 0.2% | 5 | 4.6% | 0.24 |
| Blast (Only) | 1,260,186 | 3.1% | 524 | 6.8% | 18 | 16.7% | 2.17 |
| Depth (Only) | 3,533,154 | 8.7% | 94 | 1.2% | 3 | 2.8% | 0.14 |
| Het (Only) | 2,457,194 | 6.1% | 125 | 1.6% | 1 | 0.9% | 0.27 |
| Period (Only) | 2,608,391 | 6.4% | 62 | 0.8% | 3 | 2.8% | 0.12 |
| Dup-Blast (Overall) | **6,586,048** | **16.3%** | **6778** | **87.4%** | **70** | **64.8%** | **5.37** |
| Dup-Depth (Overall) | 6,661,500 | 16.5% | 2908 | 37.5% | 26 | 24.1% | 2.28 |
| Dup-Het (Overall) | 7,152,500 | 17.7% | 5387 | 69.4% | 52 | 48.1% | 3.93 |
| Dup-Period (Overall) | 7,532,530 | 18.6% | 5457 | 70.3% | 39 | 36.1% | 3.78 |
| Sum | 16,326,794 | 40.3% | 7159 | 92.3% | 85 | 78.7% | 2.29 |

Blast: duplicates defined by blast search in reference genome (65% identity and 100bp alignable length). Depth: regions with 2x sequencing depth of average in any sequenced samples, identified through genome-wide scanning with a windows size of 100bp and a stepping size of 50bp. Het: regions carry “heterozygous” alleles (a signature of mis-mapping) in any sequenced samples, identified through genome-wide scanning with a windows size of 100bp and a stepping size of 50bp. Period: regions follow the matching period defined by Kleckner [13,14] (based on reference genome), a matching period of 10~12 with at least 3 matching bases in the beginning of each period (e.g., 3H7N, 3H8N, 4H7N, etc.) were used here. The “Enrichment” was calculated as “percentage of 2:2 mutation sites in regular sexual crosses / percentage of genomic length”.

## Table S4. Overview of identified mutation clusters within duplicates among all sexual crosses.

| **Cross** | **Cluster properties** | | | | | **Mutations in clusters** | | |
| --- | --- | --- | --- | --- | --- | --- | --- | --- |
|  | **No.** | **Total size (bp)** | **Mean size (bp)** | **Median size (bp)** | **Largest size (bp)** | **Average number per cluster** | **Highest number** | **Rate per ascus (× 10^-4^)** |
| A | 116 | 96,763 | 834.2 | 476.5 | 5,574 | 6.69 | 65 | 4.02 ± 0.48 |
| B | 81 | 69,097 | 853 | 514 | 8,137 | 5.67 | 35 | 1.67 ± 0.49 |
| E | 365 | 371,037 | 1016.5 | 478 | 11,607 | 8.61 | 139 | 4.26 ± 0.10 |
| G | 216 | 174,609 | 808.4 | 383 | 6,391 | 7.1 | 81 | 4.96 ± 0.21 |

A mutation cluster was defined as having at least two mutations within 1kb in a single haploid genome. Rate of mutations given as “Mean ± SEM**”.**

## Table S5. Approximate genomic positions of putative centromeric regions.

| Chromosome | Approximate Start | Approximate End |
| --- | --- | --- |
| Supercontig_12.1 | 3736000 | 3969000 |
| Supercontig_12.2 | 1105000 | 1346000 |
| Supercontig_12.3 | 705000 | 951000 |
| Supercontig_12.4 | 894000 | 1068000 |
| Supercontig_12.5 | 932000 | 1209000 |
| Supercontig_12.6 | 2811000 | 3060000 |
| Supercontig_12.7 | 2059000 | 2275000 |

Original positions were extracted from Smith et al. [15]. Positions on Supercontig_12.7 were adjusted according to the results of BLASTn search using Neurospora crassa centromere VII region repeat DNA (NCBI GenBank ID: AF079510.1) against the NC12 reference genome.

##

## Table S6. Number of mutations per genome employing the least generous (most conservative) definition of non-duplicates.

| Crosses | Mutations per genome | | | CDS mutations per genome | | |
| --- | --- | --- | --- | --- | --- | --- |
|  | **Overall** | **C->T only** | **C->T (%)** | **Overall** | **C->T only** | **C->T (%)** |
| Regular crosses 2:2 mutations | 10.04 | 3.57 | 35.5% | 3.62 | 1.22 | 33.8% |
| Regular crosses 3:1 mutations | 0.240 | 0.208 | 86.8% | 0.031 | 0.02 | 65.3% |
| FGSC2225-originated 2:2 mutations | 2.15 | 1.10 | 51.4% | 0.985 | 0.291 | 29.5% |
| *dim2* *rid* cross 2:2 mutations | 0.34 | 0 | 0.00% | 0.085 | 0 | 0.00% |
| Asexual mutations | 0.126 | 0.032 | 25.6% | 0.081 | 0.013 | 16.0% |

Note that outside of duplicates CDS mutations are predominantly only seen in the regular sexual cross and not in RIP deficient strains, even though RIP should not affect non-duplicate sequence. Note too that the mutations are not the classical RIP C->T type. For sexual crosses the numbers are given as “mutations per genome for sexual cycle”. For asexual lines the numbers are given as “mutations per genome per day”. Least generous duplicates represent non-duplicates by all four mentioned definitions. “C->T only” stands for C->T or G->A mutations only.

## Table S7. Cross species estimates of mutation rates and related parameters.

| N | Species | μ per bp | Class | Het | P | Ne (Lynch estimate) | Ne (re-estimate) | Genome Size | CDS % | μ per CDS |
| --- | --- | --- | --- | --- | --- | --- | --- | --- | --- | --- |
| 1 | *Apis mellifera* | 3.40E-09 | m | NA | 4 | NA | NA | 262 | 28.968 | 0.0985 |
| 2 | *Arabidopsis thaliana* | 6.95E-09 | m | 0.0008 | 4 | 288669 | 28800.0184 | 119.7 | 42.07 | 0.29239 |
| 3 | *Caenorhabditis briggsae* | 1.33E-09 | m | 0.00142 | 4 | 267380 | 267296.855 | 104 | 24.1 | 0.032 |
| 4 | *Caenorhabditis elegans* | 1.45E-09 | m | 0.00314 | 4 | 541379 | 543084.596 | 100.3 | 25 | 0.03625 |
| 5 | *Daphnia pulex* | 5.69E-09 | m | 0.0188 | 4 | 826011 | 841837.082 | 250 | 30.167 | 0.17165 |
| 6 | *Drosophila melanogaster* | 5.17E-09 | m | 0.01783 | 4 | 863020 | 877837.53 | 168.7 | 23.17 | 0.11967 |
| 7 | *Heliconius melpomene* | 2.90E-09 | m | 0.024 | 4 | 2068966 | 2119841.72 | 273.79 | 39.1 | 0.11339 |
| 8 | *Homo sapiens* | 1.35E-08 | m | 0.00114 | 4 | 21091 | 21135.2053 | 3300 | 36.45 | 0.49253 |
| 9 | *Mus musculus* | 5.40E-09 | m | 0.00383 | 4 | 177315 | 177996.542 | 2717 | 35.47 | 0.19154 |
| 10 | *Oryza sativa* | 7.10E-09 | m | 0.0015 | 4 | 52817 | 52896.2458 | 389 | 101.331 | 0.71945 |
| 11 | *Pan troglodytes* | 1.2E-08 | m | 0.00138 | 4 | 28750 | 28789.7298 | 3524 | 37.19 | 0.44628 |
| 12 | *Pristionchus pacificus* | 2E-09 | m | 0.014 | 4 | 1750000 | 1774847.87 | 169.7 | 29.66 | 0.05932 |
| 13 | *Chlamydomonas reinhardtii* | 3.80E-10 | se | 0.0329 | 2 | 43312270 | 44762148.4 | 111.101 | 39.157 | 0.01487 |
| 14 | *Paramecium tetraurelia* | 1.94E-11 | se | 0.0079 | 4 | 101804124 | 102614781 | 72.095 | 56.802 | 0.0011 |
| 15 | *Plasmodium falciparum* | 2.08E-09 | se | 0.00144 | 2 | 345489 | 346653.027 | 22.85 | 12.06 | 0.02513 |
| 16 | *Saccharomyces cerevisiae* | 2.63E-10 | se | 0.0041 | 2 | 7782840 | 7826766.55 | 12.463 | 8.709 | 0.00229 |
| 17 | *Schizosaccharomyces pombe* | 2.17E-10 | se | 0.00604 | 2 | 13901676 | 14001620.5 | 19.63 | 7.178 | 0.00156 |
| 18 | *Trypanosoma brucei* | 1.38E-09 | se | 0.02937 | 4 | 5332244 | 5481648.18 | 26.075 | 13.152 | 0.01811 |
| 19 | *Agrobacterium tumefaciens* | 2.92E-10 | b | 0.2 | 2 | 342465753 | 428082192 | 5.674 | 4.999 | 0.00146 |
| 20 | *Bacillus subtilis* | 3.35E-10 | b | 0.041 | 2 | 61194030 | 63810250.1 | 4.286 | 3.572 | 0.0012 |
| 21 | *Burkholderia cenocepacia* | 1.33E-10 | b | 0.0657 | NA | 246992481 | NA | 7.703 | 6.741 | 0.0009 |
| 22 | *Deinococcus radiodurans* | 4.99E-10 | b | NA | NA | NA | NA | 3.284 | 2.947 | 0.00147 |
| 23 | *Escherichia coli* | 2.00E-10 | b | 0.07184 | 2 | 179600000 | 193501121 | 4.64 | 3.9 | 0.00078 |
| 24 | *Helicobacter pylori* | 1.90E-09 | b | 0.15057 | 2 | 39665437 | 46647380.3 | 1.655 | 1.52 | 0.00288 |
| 25 | *Mesoplasma florum* | 9.78E-09 | b | 0.0209 | 2 | 1068507 | 1091315.66 | 0.79 | 0.734 | 0.00718 |
| 26 | *Mycobacterium smegmatis* | 5.27E-10 | b | NA | NA | NA | NA | 6.99 | 6.51 | 0.00343 |
| 27 | *Mycobacterium tuberculosis* | 1.95E-10 | b | NA | NA | NA | NA | 4.405 | 4.03 | 0.00079 |
| 28 | *PseudomoNAs aeruginosa* | 7.92E-11 | b | 0.03329 | 2 | 210164141 | 217401435 | 6.529 | 5.902 | 0.00047 |
| 29 | *Salmonella enterica* | 1.74E-10 | b | 0.1211 | 2 | 348991354 | 395936404 | 4.862 | 4.03 | 0.0007 |
| 30 | *Salmonella typhimurium* | 1.52E-10 | b | NA | NA | NA | NA | 4.86 | 4.32 | 0.00066 |
| 31 | *Staphylococcus epidermidis* | 7.40E-10 | b | 0.052 | NA | 35135135 | NA | 2.565 | 2.1 | 0.00155 |
| 32 | *Thermus thermophilus* | 1.38E-10 | b | 0.063 | 2 | 228426396 | 243608185 | 2.13 | 2.07 | 0.00029 |
| 33 | *Vibrio cholerae* | 1.15E-10 | b | 0.11 | NA | 478260870 | NA | 3.945 | 3.436 | 0.0004 |
| 34 | *Vibrio fischeri* | 2.08E-10 | b | NA | NA | NA | NA | 4.274 | 3.72 | 0.00077 |
| A | *Neurospora crassa -* mitotic | 6.03E-10 | se | 0.01476 | 2 | 1799890 | 12422157.0 | 38.64 | 14.51 | 0.0067 |
| P | *Neurospora crassa -* Lynch | 4.10E-09 | se | 0.01476 | 2 | 1799890 | 1826966.02 | 38.64 | 14.51 | 0.05949 |
| S | *Neurospora crassa -* sexual | 0.00000338 | m | 0.01476 | 2 | 1799890 | 2216.14221 | 38.64 | 14.51 | 5.34 |

N refers to the number (or letter) as they data appear in Fig. 2a-c. Column three is the mutation rate per base pair either per sexual genome for multicell species or per mitotic division for single cell species. The relevant classification is given in the next column wherein m= multicell, se=single cell eukaryote, b= bacteria. Het is heterozygosity is as defined by Lynch et al [2]. P is the ploidy adjustment (see Materials and Methods). We provide two *Ne* estimations, the original compendium [2] and our reanalysis given two different mutation rates in *Neurospora* – mitotic and sexual. Genome size is in MB, CDS % is the percentage of the genome that is CDS and the final column is then the average number of mutations in CDS each generation (sexual or mitotic). For *Neurospora* we directly estimate the latter.

## Table S8. Summary features of Dup- Cl, One, and Zero regions

| **Regions** | **Dup-Cl** | **Dup-One** | **Dup-Zero** | **Non-Dup** |
| --- | --- | --- | --- | --- |
| Total length (Mb) | 2.11 (2.08) | 2.32 (2.06) | 2.16 (1.07) | 31.1 |
| No. of 2:2 mutations per ascus | 106 | 17.9 | 0 | 31 |
| No. of blocks | 241 (215) | 401 (283) | 3,826 (430) | - |
| Average length (bp) | 8,768 (9,687) | 5,774 (7,273) | 5,64 (2,481) | - |
| Median length (bp) | 6,400 (7,000) | 4,000 (5,400) | 200 (1,400) | - |
| GC content* (%) | 28.0 ± 2.8 | 28.9 ± 3.8 | 32.3 ± 9.0 | 51.6 ± 4.2 |
| Best identity** (%) | 85.5 ± 4.2 | 84.4 ± 4.8 | 85.3 ± 5.6 | - |
| Copy numbers | 25.7 ± 29.3 | 23.7 ± 28.2 | 21.7 ± 25.4 | - |

Dup-Cl: duplicates (defined by BLAST search) with clustered mutations; Dup-One: duplicates with only singleton mutations; Dup-Zero: non-mutated duplicates. Numbers in parentheses represent results after filtering for canonical RIP threshold, i.e., ≥ 80% identity and ≥ 400bp alignable DNA length.

*GC content, Best identity and copy numbers are given in mean ± standard deviation; ** Best identity and copy numbers were estimated after dissecting each block into 200bp windows

## Table S9. Windows with putative Hi-C interactions.

| **Targets**  **Sources** | **Dup-Cl** | **Dup-One** | **Dup-Zero** | **Non-Dup** | **Total assessed windows** |
| --- | --- | --- | --- | --- | --- |
| Dup-Cl | 157 (44.6%) | 96 (27.3%) | 19 (5.4%) | 26 (7.4%) | 352 (100%) |
| Dup-One | 141 (42.7%) | 107 (32.4%) | 21 (6.4%) | 9 (2.7%) | 330 (100%) |
| Dup-Zero | 49 (21.8%) | 43 (19.1%) | 17 (7.6%) | 47 (20.9%) | 225 (100%) |
| Non-Dup | 147 (9.7%) | 114 (7.5%) | 85 (5.6%) | 478 (31.6%) | 1,512 (100%) |

The numbers given here are the number of windows (10kb in size) in a certain source which have a “observed versus expected ratio (log2)” ≥ 2.25 in one contain type of targets. Windows contain duplications with identity between 65%~80% were considered as ambiguous and were thus discarded in this analysis. The proportion of each number is given within parenthesis. Source data from Galazka, J. M. et al. [12].

# Supplementary References

1. Lynch M. Evolution of the mutation rate. Trends in Genetics. 2010;26:345–52.

2. Lynch M, Ackerman MS, Gout J-F, Long H, Sung W, Thomas WK, et al. Genetic drift, selection and the evolution of the mutation rate. Nat Rev Genet. 2016;17:704–14.

3. Koh LY, Catcheside DEA. Mutation of msh-2 in Neurospora crassa does not reduce the incidence of recombinants with multiple patches of donor chromosome sequence. Fungal Genetics and Biology. 2007;44:575–84.

4. Chary P, Dillon D, Schroeder AL, Natvig DO. Superoxide Dismutase (Sod-1) Null Mutants of Neurospora Crassa: Oxidative Stress Sensitivity, Spontaneous Mutation Rate and Response to Mutagens. Genetics. 1994;137:723–30.

5. Watters MK, Stadler DR. Spontaneous Mutation during the Sexual Cycle of Neurospora Crassa. Genetics. 1995;139:137–45.

6. Dillon D, Stadler D. Spontaneous Mutation at the Mtr Locus in Neurospora: The Molecular Spectrum in Wild-Type and a Mutator Strain. Genetics. 1994;138:61–74.

7. Sakai W, Wada Y, Naoi Y, Ishii C, Inoue H. Isolation and genetic characterization of the Neurospora crassa REV1 and REV7 homologs: evidence for involvement in damage-induced mutagenesis. DNA Repair. 2003;2:337–46.

8. de Serres FJ, Brockman HE. Comparison of the spectra of genetic damage in formaldehyde-induced ad-3 mutations between DNA repair-proficient and -deficient heterokaryons of Neurospora crassa. Mutation Research/Reviews in Mutation Research. 1999;437:151–63.

9. Selker EU. Premeiotic Instability of Repeated Sequences in Neurospora Crassa. Annual Review of Genetics. 1990;24:579–613.

10. Galagan JE, Calvo SE, Borkovich KA, Selker EU, Read ND, Jaffe D, et al. The genome sequence of the filamentous fungus Neurospora crassa. Nature. 2003;422:859–68.

11. Margolin BS, Garrett-Engele PW, Stevens JN, Fritz DY, Garrett-Engele C, Metzenberg RL, et al. A methylated Neurospora 5S rRNA pseudogene contains a transposable element inactivated by repeat-induced point mutation. Genetics. 1998;149:1787–97.

12. Galazka JM, Klocko AD, Uesaka M, Honda S, Selker EU, Freitag M. Neurospora chromosomes are organized by blocks of importin alpha-dependent heterochromatin that are largely independent of H3K9me3. Genome Res. 2016;26:1069–80.

13. Gladyshev E, Kleckner N. Direct recognition of homology between double helices of DNA in Neurospora crassa. Nature Communications. 2014;5:3509.

14. Gladyshev E, Kleckner N. Recombination-Independent Recognition of DNA Homology for Repeat-Induced Point Mutation (RIP) Is Modulated by the Underlying Nucleotide Sequence. PLOS Genet. 2016;12:e1006015.

15. Smith KM, Phatale PA, Sullivan CM, Pomraning KR, Freitag M. Heterochromatin Is Required for Normal Distribution of Neurospora crassa CenH3. Molecular and Cellular Biology. 2011;31:2528–42.
