# Supplementary material for: Repeat-induced point mutation in Neurospora crassa causes the highest known mutation rate and mutational burden of any cellular life
Source: Genome Biol. 2020 Jun 16;21:142. doi: 10.1186/s13059-020-02060-w (PMC7296669; doi:10.1186/s13059-020-02060-w)
Supplement: Supplementary file 3 — Additional file 3. Review history. [file 13059_2020_2060_MOESM3_ESM.docx]

**Review History**

**First round of review:**

**Reviewer 1:**

Wang et al. presented a detailed and solid study on the model fungus Neurospora crassa with repeat induced point mutations for silencing repeated genomic regions, and the detailed molecular mechanism for this phenomenon is not totally clear.

The study is carefully designed and analyzed with precautions. While the authors need to make the following revisions before it goes to the next step.

1. On page 14 about the drift-barrier hypothesis. The authors exaggerated the problems with the drift-barrier hypothesis for the overall set of data we have now. There is no way that the mutation rate estimates have such high sampling error that for example microbes are artifactually placed where humans are. Any hypothesis has its limits, but the situation is overstated in this paper. This exact statistical artifact problem was acutally pointed out before (Sung et al. PNAS 2012, Supporting information , SI Text). The statistical artifact brought up is correct, but only to an extent. If both pi and the mutation rate were estimated with tiny errors, there is no artifactual scaling whatsoever. The magnitude of the effect depends on the sampling variance of both pi and u. The slope of a regression is equal to the ratio of the covariance (x,y) and variance (x). Let y be pi divided by x = u.  If there is no error in u then the the estimate of Ne (y) is noisy, but the covariance is unaffected because one is always dividing by the correct u. And if there is no error in u, then variance(x) is correct too. Any serious artifact depends on having inaccurate estimates of u, and even here it is complicated, as this increases the variance of u, but will also tend towards a negative covariance. pi in N. crassa needs a good estimate-there is probably no available estimation using large-scale and whole-genome population data to date. The authors need to tone down their overstating of the statistical artifact.

2. The authors need to further annotate the mutations falling in repeated genomic regions in Data S2 sheet, additional file 2: add the repeat types (simple sequence repeats, very rarely transposable elements, other types of repeats) to the "relation to genic regions" column.

3. Details about avoiding contaminations during experiments in this filamentous fungus with spores should be introduced, as contaminations are quite easy to happen especially in the repeated asexual cycles, for example during conidia collection.

4. A lot of editorial errors need to be cleaned up. For example, the formats of literatures in the References section are not consistent; grammatical errors-"these the" line 16, page4/"using a criteria" line 23, page 9/"our results also suggests" line 45, page 15/"two mating strain" line 36, page 16/"This way we could" line 1, page 20/"sexual and asexual cycle" line 9, page 21/"the GC content was found to increased in" page 4, additional file 1; Fig. 2 definitely needs optimization for visualization, look at those messy overlapping numbers...

5. Additional file 1, Figure S1, the asexual bars do not seem to add up to 1, please double check.

6. Additional file 2, Data S1, did the authors confuse MQ with Phred sequencing quality score (Q)? GATK's MQ is calculated based on MAPQ of reads supporting reference vs. that supporting the alternative base, while the formula given was for quality score. Check

**Reviewer 2:**

The authors have analyzed de novo mutations in the progeny of crosses between different strains of the fungus Neurospora crassa. They have found that lots of RIP-like mutations occurred in the apparently non-repetitive (as defined by BLAST searches) intergenic regions. The authors have therefore claimed that "Neurospora has the highest mutation rate and mutational burden of any non-viral life". This claim may be fundamentally flawed, because RIP can recognize weak interspersed homology, while BLAST cannot. Thus, there is a possibility that degenerate (previously RIPed) repeats can still be recognized by RIP but not by BLAST. The authors need to consult a previously published study that experimentally observed RIP between repeats that shared only 37% homology, PLoS Genet. 2016 May 5;12(5):e1006015. Such repeats cannot be identified by BLAST.

The authors are also urged to consult and cite a previous study, Genetics. 1995 Jan;139(1):137-45, that had addressed the very question the authors are addressing in their manuscript. That study did not find elevated levels of RIP-like mutation in the coding sequence of the mtr gene, in agreement with the current finding. Interestingly, that previous study found other types of mutations that were associated specifically with the sexual phase. The authors need to analyze their data in the light on this previous report.

The most interesting aspect of this study is the claim that RIP-like mutations still occur in the absence of RID and DIM-2, albeit at very low level. The authors may wish to focus on exploring this original result in more detail.

**Response to reviewers:**

Reviewer #1: Wang et al. presented a detailed and solid study on the model fungus Neurospora crassa with repeat induced point mutations for silencing repeated genomic regions, and the detailed molecular mechanism for this phenomenon is not totally clear.

The study is carefully designed and analyzed with precautions. While the authors need to make the following revisions before it goes to the next step.

1. On page 14 about the drift-barrier hypothesis. The authors exaggerated the problems with the drift-barrier hypothesis for the overall set of data we have now. There is no way that the mutation rate estimates have such high sampling error that for example microbes are artifactually placed where humans are. Any hypothesis has its limits, but the situation is overstated in this paper. This exact statistical artifact problem was acutally pointed out before (Sung et al. PNAS 2012, Supporting information , SI Text). The statistical artifact brought up is correct, but only to an extent. If both pi and the mutation rate were estimated with tiny errors, there is no artifactual scaling whatsoever. The magnitude of the effect depends on the sampling variance of both pi and u. The slope of a regression is equal to the ratio of the covariance (x,y) and variance (x). Let y be pi divided by x = u. If there is no error in u then the the estimate of Ne (y) is noisy, but the covariance is unaffected because one is always dividing by the correct u. And if there is no error in u, then variance(x) is correct too. Any serious artifact depends on having inaccurate estimates of u, and even here it is complicated, as this increases the variance of u, but will also tend towards a negative covariance. pi in N. crassa needs a good estimate-there is probably no available estimation using large-scale and whole-genome population data to date. The authors need to tone down their overstating of the statistical artifact.

Response: We agree and have rewritten the section. The point that the null expectation isn’t clear and that an alternative means to estimate Ne would be valuable, remain valid. We now also add that for both fig 2b and fig 2c, the real negative correlation is significantly more negative than the simulants. We should have acknowledged that Sung et al noticed the problem with the spurious negative correlation, an oversight we now correct.

2. The authors need to further annotate the mutations falling in repeated genomic regions in Data S2 sheet, additional file 2: add the repeat types (simple sequence repeats, very rarely transposable elements, other types of repeats) to the "relation to genic regions" column.

Response: Thank you for this suggestion. We have added the repeat types to the “relation to genic regions” to Additional file 2, Data S2. The following descriptions are also added: “Types of repeats are classified using RepeatMasker (http://www.repeatmasker.org/, version open-4.0.7) based on the repeat library from RepBase (https://www.girinst.org/repbase/, version 20170127). Repeats that could not be classified to any known types are marked as ‘Unclassified’.”

3. Details about avoiding contaminations during experiments in this filamentous fungus with spores should be introduced, as contaminations are quite easy to happen especially in the repeated asexual cycles, for example during conidia collection.

Response: We are confident contamination will not affect our results. We have added more experimental details in Methods to clarify why:

“To avoid contamination, all culture media, as well as plates, were autoclaved at ~121°C for over 25 minutes in high-pressure steam sterilizer (TOMY SX-500) before each experiment. Subsequent procedures were conducted on clean benches which had been sterilized using ultraviolet immediately prior to the experiment”.

“Though we were especially cautious during every step of the experiments, we cannot be 100% sure that no contamination was present in the sequenced DNA. However, we note that contamination should not confound our mutation calling for several reasons. First, if they have a chance to be mapped to the reference genome contaminants will generally give false “heterozygous variant calls”, but all the mutations that we employ are homozygous. “Homozygous calls” from contamination would only happen in the rare incidence in which the contaminant could be mapped to regions with large deletions in the sequenced strain (i.e., the contaminant is more similar to the reference genome than the sequenced strain). Second, contaminants usually give spurious alignments with clipped read ends as they tend not to map well to the reference genome, such alignments being discarded during mutation calling. Third, contaminants are expected to be randomly distributed among all collected samples, but mutations are either 2:2 or 3:1 segregated and generally present within a single ascus.”

4. A lot of editorial errors need to be cleaned up.

For example, the formats of literatures in the References section are not consistent; We adjusted the formats of literatures in the References to make them consistent.

grammatical errors-"these the" line 16, page4/. We rewrote the sentence for clarity
"using a criteria" line 23, page 9/. Criteria replaced with criterion
"our results also suggests" line 45, page 15/ suggests change to suggest
"two mating strain" line 36, page 16/ strain->strains
"This way we could" line 1, page 20/rewritten
"sexual and asexual cycle" line 9, page 21/cycle->cycles

"the GC content was found to increased in" page 4, additional file 1; increased -> increase
Fig. 2 definitely needs optimization for visualization, look at those messy overlapping numbers... The Fig. 2 has been optimized.

5. Additional file 1, Figure S1, the asexual bars do not seem to add up to 1, please double check.

Response: We have double checked this figure and the asexual bars do add up to 1. The fractions are 0.250, 0.234, 0.156, 0.172, 0.047, and 0.141 for the six asexual bars from left to right, respectively. Original numbers used to calculate these fractions are provided in Additional file 1, Table S1. To further ensure this plot is correct, we regenerated this plot using the original data and added horizontal guide lines in this figure for easier reading.

6. Additional file 2, Data S1, did the authors confuse MQ with Phred sequencing quality score (Q)? GATK's MQ is calculated based on MAPQ of reads supporting reference vs. that supporting the alternative base, while the formula given was for quality score. Check.

Response: We use “MQ” here to represent the MAPQ (mapping quality) given by BWA, which is phred-scaled as described in the SAM format file (http://bio-bwa.sourceforge.net/bwa.shtml). We agree that this abbreviation can easily be confused with the MQ (RMSMappingQuality as explained by the reviewer) in GATK. We changed this abbreviation to MAPQ and clarified the definition as “Mapping quality (phred-scaled) given by BWA”.

Reviewer #2: The authors have analyzed de novo mutations in the progeny of crosses between different strains of the fungus Neurospora crassa. They have found that lots of RIP-like mutations occurred in the apparently non-repetitive (as defined by BLAST searches) intergenic regions.

Response: With due respect this isn’t what we claim. The only time we really attempt to infer what is going on in non-RIP domains we employ an extremely liberal definition (not just BLAST). We observe that there is still a high mutation rate outside of duplicate, but importantly not in a RIP-like manner (i.e. not disproportionately C->T). We think we might not have been clear enough so now when considering non-duplicate CDS mutations we write:

“Even with the most conservative non-duplicate definition (i.e., non-duplicate by any of four definitions, see Materials and Methods) the CDS mutation rate is 3.62 per genome per cycle for sexual 2:2 mutations in non-duplicate sequence, only ~30% of the 3.62 mutations being C->T mutations (Additional file 1: Table S6). This frequency is comparable to that seen in asexual lines (23%) and lower than that in the RIP double knockout (38%) indicative of not being directly affected by RIP. ”

The authors have therefore claimed that "Neurospora has the highest mutation rate and mutational burden of any non-viral life". This claim may be fundamentally flawed, because RIP can recognize weak interspersed homology, while BLAST cannot.

Response: The referee has misconstrued that basis of this claim. This claim is independent of the definition of RIP or nonRIP – it is simply observational. To be clear, we start by estimating from parent offspring sequencing the mutation rate overall and the rate in CDS. Only after this do we attempt to ask whether RIP might be the cause. Thus, our claim that "Neurospora has the highest mutation rate and mutational burden of any non-viral life" is independent of RIP classification and cannot be flawed because RIP can recognize interspersed homology.

Thus, there is a possibility that degenerate (previously RIPed) repeats can still be recognized by RIP but not by BLAST. The authors need to consult a previously published study that experimentally observed RIP between repeats that shared only 37% homology, PLoS Genet. 2016 May 5;12(5):e1006015. Such repeats cannot be identified by BLAST.

Response: We very much agree with the referee that in calling some mutations as nonRIP simply because BLAST doesn’t consider the sequences to be repetitive would be unsafe. But this is not what we did. We think the referee might have misunderstood our claims and their basis. Indeed, for the reason outlined by the referee, where we really wanted to isolate domains likely not subject to RIP (i.e to consider whether mutation in CDS might be owing to RIP in classical form) we a) consider non-duplicate as sequence described as non-duplicate by every method of duplicate calling, not just BLAST (and we did indeed cite the above paper and employ the method), b) consider the form of the mutations (we noted that the raised rate is not associated with RIP-like events ie not C->T.) but c) also note that the raised rate is not present in the double RIP mutant. For these reasons we suggest that the raised CDS rate is RIP-associated (owing to observation c), but not classical RIP (observations a and b). To further clarify the argument, we now additionally note that outside of duplicates (very broadly defined) the C->T rate is about the same as in asexual lines and lower than in the double knockout (see above). This we hope clarifies that these mutations cannot be ascribed directly to the action of RIP.

The authors are also urged to consult and cite a previous study, Genetics. 1995 Jan;139(1):137-45, that had addressed the very question the authors are addressing in their manuscript.

Response: Thank you for this suggestion. We discussed and cited this paper in the supplement (ref 5) along with the other prior single gene estimates of the mutation rate in Neurospora. We now additionally note in the introduction that this paper provides evidence for nonRIP sexual mutations. We use this as direct motivation for the use of the knockout lines (comparing sexual with sexual). We also return to this paper now in the discussion to emphasise that their analysis on a single gene by necessity underestimates the effects of RIP genome wide. We also note that our data suggests very limited scope for mutations particular to the sexual cycle that are not RIP associated (98% are associated with RIP according to the double knockout) and that our data agrees with this paper in finding no good evidence for meiotic mutations (we see none in the RIP double knockouts).

That study did not find elevated levels of RIP-like mutation in the coding sequence of the mtr gene, in agreement with the current finding. Interestingly, that previous study found other types of mutations that were associated specifically with the sexual phase. The authors need to analyze their data in the light on this previous report.

Response: We agree that we should mention that others observe different mutational profiles through the sexual phase. We now raise this in the introduction and in the discussion. We note that if 98% of sexual mutations are RIP associated – as the double KO indicates - this leaves only very minor room for sexual non RIP mutations. This prior paper also considered indels which we do not.

The most interesting aspect of this study is the claim that RIP-like mutations still occur in the absence of RID and DIM-2, albeit at very low level. The authors may wish to focus on exploring this original result in more detail.

Response: While we disagree that this is the most interesting observation (it isn’t one we wish to bring much attention to), we are nonetheless grateful for this suggestion. We have, as requested, expanded consideration and included a new subsection:

The double knockout cross is largely (but possibly not entirely) devoid of RIP activity
If we take the profile of mutations through the asexual cycle as a null, we can ask whether the double knockout shows evidence of residual RIP activity, based on the class of mutations and their clustering. Analysis of the form and location of the mutations suggest that the double knockout cross is largely, but possibly not entirely, devoid of RIP activity.

Of the 13 mutation sites, only 5 are RIP-type C->T changes, the remaining 8 are non-RIP C->T changes (Additional file 2: Datasheet S2). Assuming that the asexual lines provide a fair comparator, we would expect 3.042 C->T and 9.958 non C->T, which is not significantly different from what is observed (with expectation drawn from asexual proportions, χ2 with Yate’s correction = 0.91, df=1, P>0.05). This suggests little or no residual RIP activity. Overall 9 of 13 (69.2%) of mutation sites were found in repeat regions, compared with 44.4% in asexuals (Additional file 2: Datasheet S2) (with expectation drawn from asexual proportions, χ2 with Yate’s correction = 2.41, df=1, P>0.05). However, this disguises the observation that all the C->T changes are in repeat regions while, of the eight others, four are in repeat regions. Most suggestively, four of the “C->T” mutations sites were in a cluster, this being the only mutation cluster found in the mutant cross. This is indicative of a small residual degree of RIP even in the double knockout, worthy of further scrutiny.

**Second round of review**

**Reviewer 2**

I must review the code (written in Perl), to make sure the authors understand the notion of interspersed homology, at least in the form presented in my papers.

Let's say below is my mock genome that contains a pattern on interspersed homology

>mock_genome
AGGACAGAACGCAGCAGCAGAGCTTCTCTCTCCGGTGCGCGGCTTTCTGAAGATAGTTAT
TGTCCGGAGCGACAGTTGATGGTGGATCCTGCGGCTATAGGGAGGTCGTTGCAGCGACGC
CGGGTTCTGGAGGAGCTCATCAACACGGAAGAGAGTTATATTGGAGATGTGCGCTTCCTG
ATGAATGTATGTCGCAGCTTCATCCCGTCCTCTTTCACTGTCTCTGACTAGTCTACCCCT
AACCTAGGTTTATGTTACCATTCTTGCTTCGCTTCCTACCTTGCCAGCCAGCCTTCGCTC
TTCTATCAATCAAAATCTCAATGACATTGTTGAGCTGCATGAGGAGATGTTAGGCGACCT
TCATAGAGCTATTCCTCACTCTGAGTATACACAGCTCAAATTCCCACTGCAAGTTCCTTC
GGCCGGCTCGGGGTTTCGTGGTCATCAGCGATGGAGAAGTCTGGATGCAGTACCAGAAGA
CAAGGATGGCATCTCATGGCTTCGTGAAGTTCCCGGCATGCTTGCCGACCCACAGATTGC
TGCTGAAGTTGCCAAGATATTCAGCAAGAAGATGAACCGATTTTTCATCTACGAAGAGTA
TGGTGCCAAGTACGAGCTGATGATCAAAGATGTTGCTTCTGCTCACAGGACAATGCCTGC
ATGGCAATCATATCAAAAGGGCATTGAGATTCTGGCTTCCTCTCTTAGCTCGGCTAACAG
TCAGGACGACAAACAGAGGAAAAGTCTCACGATTGGGGATCTGCTCATGAAGGTAGGCGA
TATATCGCACAAAAACACGCGGATGTATGCTAACAAGGACCAGCCAATCCAGCGTGTGTG
CAAATACCCATTGCTCTTCGCCGAGTTGCTAAAACACACTCCTGAGGCAGACTGCCCCTA
TTCTCATATGGAAATCGAAAACACGCTTATTCGGTTGCGTGAGGCTACTGCCGAAATCAA
CAAAGCTACGGATGATTCTAAAATCAAGTCCACTCTGGAGAAAACATGGATCCTCCAAGA
CCGTCTGGTGTTTCCTAATCAGCAACTGGATGCAGCTTCAAAGAATCGCATTCGATCATT
CGGCCATATCCAACTGTGCGGAGCTCTGCATGCCTGTTGGCAGACAAAAGAAGGCGTCAA
TGGGCAGTACATGGTTGCTCTACTCTACAAGGAATGGTTCTGTCTAGCTACAGCTAGTAG
GACTGACCAGATATACACGATCCAAGCTTGCATTGCTTTGAGCAACGTCAAGTTGGAAGA
AGTGGACAACGGTCGAGGTATGACATTGGCTCTGTCAGATGTCTTCATCCGTGTGAACCT
CGTGCTGACGTATCTATAGGCCTTCAATGTCACACCGCGCCATATTCCTGGAAACTCGTG
TTTCTCTGCGACCACCAGTTGTACGAGATCATCTTGACAGCATGCACGCCGAAAGAGGAG
GTGGAATGGCGTACACGCCTCGGAAACCAACAGACCACCGAGGGCCAAGACCAGATGCAA
AGGTAGTGTGGCACGGTATTCGCTGTGGAAGCCGTCCGCAATCTTGTCCCTTATATCATG
AAGCCGACTGACGAGTGCTCAAACGATATCCACCCTAATCCATTATCGAGTGTCTGACTT
GACTAGCTGACAACTGTCACATGGCTAGAACCACACCATACAAATCGAGTGTTACGTGTG
AAAGTCCCCTGTATGCCGCTCACGATAGAATCTACGTGCCTCTACATTCGATCTTGTTTG
CACCCTTAGCCCTGTATGAGCCCTTAGGCTCGGTCCGGGAACTCCAATGTTTGCTCGTGA
CGGCATCTCCTCGGTTCTGTCGCCGATTTAGACTTAGCAGTGTTGAATGACCCTTATCCT
AGTGCCGGCTTCCAGAGTCTCAAGTAGGACAGGCTGAGTATTTAAGTACCAACACATAAA
GGCACCAAACGGGACGACAACTCAGATATCCTGGGTGTAATCGGAGCTCTCGCCACTGAT
TCTGGACATAGGTTCAAATTCATATGATCGCTATGGCCCAACCAACGATGTTATTATTTT
GAACTGAGTGTATCGAATACGGTCTTCGAACTATCCACGAAACGGTCACTATCGGTCCTA
TCAGCTGTCGTAACTTGGCATGTCTCGGTCTGTCAAGGTAGCTAGGCACTAAATAATGCT
CTGGACGAGGGCTCACTCTCAGATTCGAGAGTGGGCCAGTGGCCTTCCTATACTTAATCA
CGCCGACCGGGGTAGGAGCTCGCCGCTCTTATAAATGGACTACAAAATGCTTTACAACGA
GTGGAGCCACTGGCTAGAGCGACCACTCGCTCGTTCTTGCCACAACCGTCAGTCGTAAGA
CAATGGGTGTTTGAGAGATTTCGATCCAGGTAAAGTTGACGTTGACTACAGTTGCATGGG
CATTCAGCCTATCATCTTGTCGTAGCTCGGAAATTTGAACTTCCTTACGTGTCCCATCGG
TGCTATTACTATGACATCTCCGGAACTGTCGGTAAGAGAGTTCCACAAGATGTGTTGCGA
CACAGACAAGTTAATGGCACAGAGCGGTTCTGCTTGACTTAAGTTAACACATCGCGATCC
AGGCTGGGGTGTACTTCCACTTGCTAACGGAAGCTGACATGCAACACTGCTGCTCGTGCC
CTTTCAGGCTGGCTGTGCAAGCTAATACGCAATGGAGTTAGTCTATGCTTATCTACTTAG
TCGCAGTCAGGACGTGGAGATTATTAGCTGCCGATTGGGGAGGCTGCCAAAGCCCACCAT
AGTACCAGGAGGTTATTACTGGACTACATAACTGATGAGGCACTTGTCATAAGTGCGGAG
ACAGCTACACGTCCTACGTCTACACAACCGGCATCCGTTGTCATCTCCAACTGCATCGCA
GACGAATGCATTGTGGAGTCTGGACGAATCGGACGTGCAGTTGATTATCCGTTGATTAAG
GGATCGACTCGTTACGTGATCGCGGTAGGTCAGGAGTTTCAGGCGTTTCTTCAGGATGCC

This genome is 3000 bp, and the first 1500 bp are homologous to the last 1500 bp:

in this cases, triples are interspersed with a matching periodicity of 10 or 11 bp, which should be detected by the authors code.

I go to their depository at [https://github.com/wl13/Neurospora_mutation/tree/master/Dup-Period/perl_scripts](https://urldefense.proofpoint.com/v2/url?u=https-3A__github.com_wl13_Neurospora-5Fmutation_tree_master_Dup-2DPeriod_perl-5Fscripts&d=DwMFaQ&c=vh6FgFnduejNhPPD0fl_yRaSfZy8CWbWnIf4XJhSqx8&r=2cx_KN2CF-GjHdoDOSNnUrVR5ZNMGN9Hc_j-eNTS15k&m=gVMxE3wXQrzZWqu1mPo7aNJabh-lxadhSO_g5wsO-sQ&s=gBNSK__WA8dTm5ppEMq2cbSYETvnHAOFduHHxWqjSb0&e=) and download the first script [https://github.com/wl13/Neurospora_mutation/blob/master/Dup-Period/perl_scripts/1.chop_genome_to_group-rr.pl](https://urldefense.proofpoint.com/v2/url?u=https-3A__github.com_wl13_Neurospora-5Fmutation_blob_master_Dup-2DPeriod_perl-5Fscripts_1.chop-5Fgenome-5Fto-5Fgroup-2Drr.pl&d=DwMFaQ&c=vh6FgFnduejNhPPD0fl_yRaSfZy8CWbWnIf4XJhSqx8&r=2cx_KN2CF-GjHdoDOSNnUrVR5ZNMGN9Hc_j-eNTS15k&m=gVMxE3wXQrzZWqu1mPo7aNJabh-lxadhSO_g5wsO-sQ&s=2gHoyxsxIB35HmKx7exoRWzkNkeBmfQ8mHtP8zQDXZY&e=)

The first problem right away is that this script is using some input data stored internally, which is a VERY bad programming practice. Let's try to see if we can go around it:

First comes 'open GENOME, "</mnt/san1/usr/thw/info/neurospora/neurospora_crassa_or74a_12_supercontigs.fasta"'

I change that to 'open GENOME, "<mock_genome.fasta"' and proceed further

Then comes 'open LEN, "</mnt/san1/usr/thw/info/neurospora/chr-length.txt"'.

This is silly, since you can get the length of each config using bioperl function, no need to store contig lengths separately. From reading down below, I understand that chr-length.txt is in tab-delimited format, so I make file "mock_genome.txt" with just one line "mock_genome 3000" and change the corresponding open command to 'open LEN, "</mnt/san1/usr/thw/info/neurospora/chr-length.txt"'

now trying to execute

I type 'perl [1.chop_genome_to_group-rr.pl](https://urldefense.proofpoint.com/v2/url?u=http-3A__1.chop-5Fgenome-5Fto-5Fgroup-2Drr.pl&d=DwMFaQ&c=vh6FgFnduejNhPPD0fl_yRaSfZy8CWbWnIf4XJhSqx8&r=2cx_KN2CF-GjHdoDOSNnUrVR5ZNMGN9Hc_j-eNTS15k&m=gVMxE3wXQrzZWqu1mPo7aNJabh-lxadhSO_g5wsO-sQ&s=ADLwTY4qmw6KLLh8vBS2eVmdS_QmsDA3jtfRes5Rt6g&e=)' and I get the following:

Can not open array file.

it's issued in in this line (#19)

open ARRAY, "<p01-array-g6.txt" or die "Can not open array file\.\n";

and I have no idea what this file is (p01-array-g6.txt).

************************* end of code review

Further thoughts... let's suppose the entire approach to detect interspersed homology is flawed, the question is what else is left in the paper that can be published. Without a way to detect weak homology, one must say that the authors see residual RIP of decaying repeats. This is a trivial observation, but it's been unfortunately over-hyped as the highest mutation level of non-viral life or something like that... Slightly elevated mutation of CDS is only new claim in this manuscript. There are several problems here. First, the authors do not examine indels, which were specifically shown by the previous study to be associated with the sexual process. In this respect, the manuscript is the regression in our understanding of mutation in neurospora. Second, the authors, by doing their experiments in the lab, ignore two important issues with neurospora biology that are still critical to the conclusions of this manuscript. (A), it's a well known that the level of mutation increases in spores that emerge later in a given cross. Thus, its not clear if moderately high levels of mutation of CDS (perhaps found in late spores??) has relevance to the real world, in which both early and late spores are produced and subjected to selection. (B) it's also unclear if the strains used in this study represent the actual neurospora species in the wild. The authors themselves have found a mutation in the rid gene in one of their strains. I have no idea if the same strains could contain other mutations (=hypomorphic allles?) of DNA repair genes. In any case, the authors can only address these concerns by analyzing populations, eg summarized here [https://hal.archives-ouvertes.fr/hal-02281657/document](https://urldefense.proofpoint.com/v2/url?u=https-3A__hal.archives-2Douvertes.fr_hal-2D02281657_document&d=DwMFaQ&c=vh6FgFnduejNhPPD0fl_yRaSfZy8CWbWnIf4XJhSqx8&r=2cx_KN2CF-GjHdoDOSNnUrVR5ZNMGN9Hc_j-eNTS15k&m=3yq9WCz9qhDmoG86JpsZO3jLSTHZnhUCSjVvLygaDyY&s=KdkzhKFXxi8ZCKHbPCmtGowsbVl1gZ7FaUgRLyKwF9I&e=)

**Authors’ Rebuttal of rejection**

**Response to referee 2.**

**We were very disappointed by the quality of review provided by this referee in round 1. His core objection that our (non) failure to consider interspersed homology was “fatal” to our findings regarding observed mutation rates was wrong on all counts: it was both factually wrong and irrelevant. We did consider it, but it has nothing to do with nearly all results, including the headline results. He criticised us for not doing analysis we had done, for not presenting scripts that we had presented and for failing to cite papers we had cited. We are equally disappointed by his offhand dismissal and inaccurate reporting of our work in round 2.**

I must review the code (written in Perl), to make sure the authors understand the notion of interspersed homology, at least in the form presented in my papers.

Let's say below is my mock genome that contains a pattern on interspersed homology

>mock_genome

AGGACAGAACGCAGCAGCAGAGCTTCTCTCTCCGGTGCGCGGCTTTCTGAAGATAGTTAT TGTCCGGAGCGACAGTTGATGGTGGATCCTGCGGCTATAGGGAGGTCGTTGCAGCGACGC CGGGTTCTGGAGGAGCTCATCAACACGGAAGAGAGTTATATTGGAGATGTGCGCTTCCTG ATGAATGTATGTCGCAGCTTCATCCCGTCCTCTTTCACTGTCTCTGACTAGTCTACCCCT AACCTAGGTTTATGTTACCATTCTTGCTTCGCTTCCTACCTTGCCAGCCAGCCTTCGCTC TTCTATCAATCAAAATCTCAATGACATTGTTGAGCTGCATGAGGAGATGTTAGGCGACCT TCATAGAGCTATTCCTCACTCTGAGTATACACAGCTCAAATTCCCACTGCAAGTTCCTTC GGCCGGCTCGGGGTTTCGTGGTCATCAGCGATGGAGAAGTCTGGATGCAGTACCAGAAGA CAAGGATGGCATCTCATGGCTTCGTGAAGTTCCCGGCATGCTTGCCGACCCACAGATTGC TGCTGAAGTTGCCAAGATATTCAGCAAGAAGATGAACCGATTTTTCATCTACGAAGAGTA TGGTGCCAAGTACGAGCTGATGATCAAAGATGTTGCTTCTGCTCACAGGACAATGCCTGC ATGGCAATCATATCAAAAGGGCATTGAGATTCTGGCTTCCTCTCTTAGCTCGGCTAACAG TCAGGACGACAAACAGAGGAAAAGTCTCACGATTGGGGATCTGCTCATGAAGGTAGGCGA TATATCGCACAAAAACACGCGGATGTATGCTAACAAGGACCAGCCAATCCAGCGTGTGTG CAAATACCCATTGCTCTTCGCCGAGTTGCTAAAACACACTCCTGAGGCAGACTGCCCCTA TTCTCATATGGAAATCGAAAACACGCTTATTCGGTTGCGTGAGGCTACTGCCGAAATCAA CAAAGCTACGGATGATTCTAAAATCAAGTCCACTCTGGAGAAAACATGGATCCTCCAAGA

CCGTCTGGTGTTTCCTAATCAGCAACTGGATGCAGCTTCAAAGAATCGCATTCGATCATT CGGCCATATCCAACTGTGCGGAGCTCTGCATGCCTGTTGGCAGACAAAAGAAGGCGTCAA TGGGCAGTACATGGTTGCTCTACTCTACAAGGAATGGTTCTGTCTAGCTACAGCTAGTAG GACTGACCAGATATACACGATCCAAGCTTGCATTGCTTTGAGCAACGTCAAGTTGGAAGA AGTGGACAACGGTCGAGGTATGACATTGGCTCTGTCAGATGTCTTCATCCGTGTGAACCT CGTGCTGACGTATCTATAGGCCTTCAATGTCACACCGCGCCATATTCCTGGAAACTCGTG TTTCTCTGCGACCACCAGTTGTACGAGATCATCTTGACAGCATGCACGCCGAAAGAGGAG GTGGAATGGCGTACACGCCTCGGAAACCAACAGACCACCGAGGGCCAAGACCAGATGCAA AGGTAGTGTGGCACGGTATTCGCTGTGGAAGCCGTCCGCAATCTTGTCCCTTATATCATG AAGCCGACTGACGAGTGCTCAAACGATATCCACCCTAATCCATTATCGAGTGTCTGACTT GACTAGCTGACAACTGTCACATGGCTAGAACCACACCATACAAATCGAGTGTTACGTGTG AAAGTCCCCTGTATGCCGCTCACGATAGAATCTACGTGCCTCTACATTCGATCTTGTTTG CACCCTTAGCCCTGTATGAGCCCTTAGGCTCGGTCCGGGAACTCCAATGTTTGCTCGTGA CGGCATCTCCTCGGTTCTGTCGCCGATTTAGACTTAGCAGTGTTGAATGACCCTTATCCT AGTGCCGGCTTCCAGAGTCTCAAGTAGGACAGGCTGAGTATTTAAGTACCAACACATAAA GGCACCAAACGGGACGACAACTCAGATATCCTGGGTGTAATCGGAGCTCTCGCCACTGAT TCTGGACATAGGTTCAAATTCATATGATCGCTATGGCCCAACCAACGATGTTATTATTTT GAACTGAGTGTATCGAATACGGTCTTCGAACTATCCACGAAACGGTCACTATCGGTCCTA TCAGCTGTCGTAACTTGGCATGTCTCGGTCTGTCAAGGTAGCTAGGCACTAAATAATGCT CTGGACGAGGGCTCACTCTCAGATTCGAGAGTGGGCCAGTGGCCTTCCTATACTTAATCA CGCCGACCGGGGTAGGAGCTCGCCGCTCTTATAAATGGACTACAAAATGCTTTACAACGA GTGGAGCCACTGGCTAGAGCGACCACTCGCTCGTTCTTGCCACAACCGTCAGTCGTAAGA CAATGGGTGTTTGAGAGATTTCGATCCAGGTAAAGTTGACGTTGACTACAGTTGCATGGG CATTCAGCCTATCATCTTGTCGTAGCTCGGAAATTTGAACTTCCTTACGTGTCCCATCGG TGCTATTACTATGACATCTCCGGAACTGTCGGTAAGAGAGTTCCACAAGATGTGTTGCGA CACAGACAAGTTAATGGCACAGAGCGGTTCTGCTTGACTTAAGTTAACACATCGCGATCC AGGCTGGGGTGTACTTCCACTTGCTAACGGAAGCTGACATGCAACACTGCTGCTCGTGCC CTTTCAGGCTGGCTGTGCAAGCTAATACGCAATGGAGTTAGTCTATGCTTATCTACTTAG TCGCAGTCAGGACGTGGAGATTATTAGCTGCCGATTGGGGAGGCTGCCAAAGCCCACCAT AGTACCAGGAGGTTATTACTGGACTACATAACTGATGAGGCACTTGTCATAAGTGCGGAG ACAGCTACACGTCCTACGTCTACACAACCGGCATCCGTTGTCATCTCCAACTGCATCGCA GACGAATGCATTGTGGAGTCTGGACGAATCGGACGTGCAGTTGATTATCCGTTGATTAAG GGATCGACTCGTTACGTGATCGCGGTAGGTCAGGAGTTTCAGGCGTTTCTTCAGGATGCC

This genome is 3000 bp, and the first 1500 bp are homologous to the last 1500 bp:

in this cases, triples are interspersed with a matching periodicity of 10 or 11 bp, which should be detected by the authors code.

I go to their depository at https://github.com/wl13/Neurospora_mutation/tree/master/Dup-Period/perl_scripts and download the first script https://github.com/wl13/Neurospora_mutation/blob/master/Dup-Period/perl_scripts/1.chop_genome_to_group-rr.pl

The first problem right away is that this script is using some input data stored internally, which is a VERY bad programming practice. Let's try to see if we can go around it:

First comes 'open GENOME, "</mnt/san1/usr/thw/info/neurospora/neurospora_crassa_or74a_12_supercontigs.fasta"'

I change that to 'open GENOME, "<mock_genome.fasta"' and proceed further

Then comes 'open LEN, "</mnt/san1/usr/thw/info/neurospora/chr-length.txt"'.

This is silly, since you can get the length of each config using bioperl function, no need to store contig lengths separately. From reading down below, I understand that chr-length.txt is in tab-delimited format, so I make file "mock_genome.txt" with just one line "mock_genome 3000" and change the corresponding open command to 'open LEN, "</mnt/san1/usr/thw/info/neurospora/chr-length.txt"'

now trying to execute

I type 'perl 1.chop_genome_to_group-rr.pl' and I get the following:

Can not open array file.

it's issued in in this line (#19)

open ARRAY, "<p01-array-g6.txt" or die "Can not open array file\.\n";

and I have no idea what this file is (p01-array-g6.txt).

************************* end of code review

**Response: The code captures interspersed homology in the above sequence. Example steps as well as all intermediate/final results are given at https://github.com/wl13/Neurospora_mutation/tree/master/Dup-Period/example.**

**The “p01-array-g6.txt” file was renamed as “unit_length_combinations.txt” for ease of interpretation and is in the same folder. Nonetheless, this small error in naming a file shouldn’t have occurred. But please note these scripts were not intended to be professional level. Anyone who wants to replicate the analysis has access to the scripts and we are happy to provide any assistance. This is a standard GitHub submission.**

**Please also note that this paper is not about attempting to develop universal tools for discovering interspersed homology as, despite the repeated mischaracterizations by the referee, this is almost entirely irrelevant for the current work.**

Further thoughts... let's suppose the entire approach to detect interspersed homology is flawed

**Response: as evidenced above, it isn’t flawed,**

, the question is what else is left in the paper that can be published. Without a way to detect weak homology, one must say that the authors see residual RIP of decaying repeats. This is a trivial observation, but it's been unfortunately over-hyped as the highest mutation level of non-viral life or something like that... Slightly elevated mutation of CDS is only new claim in this manuscript.

**Response: The referee is, once again, being grossly disingenuous.**

**Previously the referee asserted that our estimate of mutation rates was “fatally flawed” because he thought we hadn’t considered interspersed homology. Now, as then, the referee is simply wrong on many fronts. The context for the paper is whether this organism has a high per bp rate and per CDS rate compared to other organisms, this being relevant in the context of mutation rate evolution. Our subsidiary question is to ask what proportion of these can be ascribed to RIP. Just about all of the conclusions don’t require any assumptions about interspersed homology: the estimates of rates per bp and in CDS per genome in the absence of RIP require nothing more than sequencing and mutation calling; the key comparison to estimate RIPs effects are the wild type v double knockout (we provide others, such as partial knockout and extrapolation from asexuals). We don’t see anything in the abstract that would change if the interspersed homology analysis was wrong, even though it isn’t. All results are new to our paper (point P in Fig 2 is the prior amalgamated estimate employed by Lynch and colleagues).**

**Moreover, the one result where we do look at interspersed homology our control is correct. This is also a minor part of one control to provide as generous a definition of rippable sequence as possible. This analysis is one sentence in the final subsection of results – and isn’t an important result. We fail to understand the motivation of the reviewer in once again grossly exaggerating the role of this minor component of a minor analysis.**

**Any reader of our paper will struggle to understand the referee’s “slightly elevated” assertion. We invite all readers of this response to look at data point S in fig 2a, b and c. You will see that the sexual cycle is a massive and obvious outlier. Fig 2a makes this especially clear as regards all mutations. Fig 2c shows CDS rate far off up the Y axis. We are happy to add two further figures to make this yet more explicit. In Fig 2d we now plot Genome Size against CDS mutation rate. Additionally, we plot CDS mutations per bp of CDS against Genome size (Fig 2e). To consider the wild types (points S) as “slightly elevated” defies comprehension.**

**The rates are as we claim the highest seen in any non-viral organism. It is unwarranted for the referee to dismiss this as “hype” as it is a) factually correct and b) central to the points we are making relevant to the comparative biology of mutation rates and theory of mutation rate evolution.**

**We now add:**

To provide a comparison controlling for genome size one can see (Fig 2d) that Neurospora’s number of CDS mutations is 2-3 orders of magnitude higher than genomes of comparable size. As this doesn’t control for variation in the amount of CDS we can also compare CDS mutations per bp of CDS (Fig 2e). Again, we see a 2-3 orders of magnitude higher rate in *Neurospora*.

There are several problems here. First, the authors do not examine indels, which were specifically shown by the previous study to be associated with the sexual process. In this respect, the manuscript is the regression in our understanding of mutation in neurospora.

**Response: The referee again misrepresents what we are doing.**

**Our core aim is to compare mutation rates between species (Fig 2) to see where Neurospora fits. In these other species the point mutation rate is what has been measured. Consequently, it is on this data that theories of mutation rate evolution have focused (see Nat Rev Genet 2016 17, 704). Were we to include indels in our analysis - where other species hadn’t - this would have made for an unfair comparison (although probably not greatly misleading given relative rates).**

**The field’s focus on point rates is sensible as a) point mutations are at least an order of magnitude more common than indels and b) point mutations can be called with some precision using WGS, where indel calling is often unsafe and highly contingent on the computational pipeline. To describe our new data as “regression” simply because it doesn’t include all possible mutations is contrary to the definition of regression. We also don’t consider epimutations, inversions, translocations etc. We did note that we didn’t analyse indels but now make the above logic clearer. We now add:**

“For consistency with prior estimates of the mutation rate (in different species) and because indel calling is prone to analysis artefacts, we restrict analysis to point mutations”.

Second, the authors, by doing their experiments in the lab, ignore two important issues with neurospora biology that are still critical to the conclusions of this manuscript. (A), it's a well known that the level of mutation increases in spores that emerge later in a given cross. Thus, its not clear if moderately high levels of mutation of CDS (perhaps found in late spores??) has relevance to the real world, in which both early and late spores are produced and subjected to selection.

**Response: The referee again both grossly exaggerates a trivial issue and (selectively) ignores the evidence.**

**Comparison with the double knockout indicates that 98% of mutations are attributable to RIP and not to differential spore timing. To suggest that this issue is “critical” to the paper is nonsensical. Even without this evidence, only if a) the mutation rate modulates by orders of magnitude from early to late and b) our sampling is grossly biased to the late spores could one even come close to considering this even a possible contender to explain extreme outlier status (see Fig 2a and 2e). The referee will know that the early late difference is modest (not orders of magnitude needed to explain outlier status) and there is nothing in our protocol to be seriously biased to late spores. We now add:**

“That 98% can be attributed to RIP through a comparison of the double knockout and wild types strongly indicates that the order of magnitude differences that we observe between Neurospora and comparable species (Fig 2) cannot be accounted for by, for example, variation in mutations in repair enzymes or modest differences in mutation rates between early and late sampled spores (and our protocol is not obviously biased one way or the other).”

(B) it's also unclear if the strains used in this study represent the actual neurospora species in the wild. The authors themselves have found a mutation in the rid gene in one of their strains. I have no idea if the same strains could contain other mutations (=hypomorphic allles?) of DNA repair genes. In any case, the authors can only address these concerns by analyzing populations, eg summarized here https://hal.archives-ouvertes.fr/hal-02281657/document

**Response: Here we follow industry best practice which is to use a number of different strains, it being thought that in many organisms different strains of the same species can have slightly different mutation rates. As sampling of wild strains finds that very few suppress RIP, lab strains and wild caught strains are comparable. Further, as the RIP double knockout has just 2% of the mutations of the wild type, we can be very confident that we know the source of nearly all “excess” mutations (see added text above).**

**We note also that mutation rate estimation requires a well-built genome and these are associated with lab maintained strains (humans are the one exception). We now add:**

As we employed numerous different strains our results are both likely to be representative of what is seen in the wild and not distorted by reliance on only one strain.

**We also now add :**

Both FGSC 2489 and 4200 are highly inbred strains, and are preferred for use as standard wild types [23]. FGSC 4200 is a progeny from the 6th backcross to FGSC 2489 (the reference genome strain) of FGSC 2490. FGSC 2225 (Mauriceville-1cA, collected from MAURICEVILLE, Texas U.S.A.) is a strain isolated from nature [24]. Strain FGSC 1363 is a morphological mutant that begins growth on agar as small colonies and sooner or later produces a flare of wild-type-appearing hyphae (with or without conidia) [25]. FGSC 3246 is a female sterile mutant [26]. From the SNP profile there is no reason to suppose that they are hyper-mutators [27]. Prior examination of RIP in nature only identified very few wild isolates that dominantly suppress RIP [28,29], suggesting similar properties of these lab strains in triggering RIP as in nature.

**In sum, the referee has invented in this and the prior round numerous factually incorrect claims, inflated trivial issues into “fatal” or “critical’ flaws when in practice the criticisms are inaccurate, wrong and exaggerated beyond all recognition. We regret that we have no confidence in this reviewer’s ability to provide an impartial professional critique.**
